# The Influence of Electrolyte Type on Kinetics of Redox Processes in the Polymer Films of Ni(II) Salen-Type Complexes

**DOI:** 10.3390/molecules27061812

**Published:** 2022-03-10

**Authors:** Danuta Tomczyk, Piotr Seliger, Wiktor Bukowski, Karol Bester

**Affiliations:** 1 Department of Inorganic and Analytical Chemistry, University of Łódź, ul. Tamka 12, 91-403 Łódź, Poland; piotr.seliger@chemia.uni.lodz.pl; 2 Faculty of Chemistry, Rzeszów University of Technology, Al. Powstańców W-wy 6, 35-959 Rzeszów, Poland; wbuk@sd.prz.edu.pl (W.B.); bester_k@prz.edu.pl (K.B.)

**Keywords:** nickel complex, Schiff base ligand, electropolymerization of complexes, kinetics, modified electrode

## Abstract

Electrodes modified with polymers derived from the complexes [Ni(salcn)], [Ni(salcn(Me))] and [Ni(salcn(Bu))] were obtained in order to study the kinetics of electrode processes occurring in polymer films, depending on the thickness of the films, the type of electrolyte and the solvent. FTIR and EQCM methods were used to determine the type of mass transported into polymer films during anode processes and the number of moles of ions and solvent. The rate of charge transport through films was determined by the cyclic voltammetry method, by the quantity *cD*^1/2^. It was shown that the charge transport was determined by the transport of anions. The kinetics were most efficient for *poly*[Ni(salcn(Bu))] modified electrodes, obtained from TBAPF_6_ and working in TBAClO_4_ and TBABF_4_. It was also shown that a solvent with a higher DN value and lower viscosity (MeCN) facilitated the transport of the charge through polymer films.

## 1. Introduction

Electrodes modified with films derived from nickel complexes with salen and its derivatives have been investigated, among others, due to the possibility of using them in electrocatalysis and electroanalysis [1,2,3,4,5,6,7,8] and as material for energy storage [9,10,11,12]. The structural, photochemical [13] and catalytic [14,15] properties of the complexes and electropolymerization mechanisms [16,17,18,19,20,21,22,23,24,25,26,27,28,29] have been analyzed and kinetic studies have been conducted [3,23,25,29]. Complexes with free 5,5′ positions undergo electropolymerization [16,17,18,19,20,21,23,24,25,26,27], while complexes substituted in these positions do not form polymer films on the electrode surface [30,31,32]. Modified electrodes are obtained in solvents that do not have coordinating properties [16,17,18,19,20,21,22,23,24,25,26,27,28,29], usually by anodic electropolymerization [16,17,18,19,20,21,23,24,25,26,27]. Solvents with coordinating properties occupy axial positions in the coordination sphere of the central ion, which prevents polymerization [33,34]. Studies of the electropolymerization mechanism show that the ligand is oxidized in some of the complexes [16,17,18,19,20,21,23,24,25,26,27], in others the central ion [28,29] and, in still others, both the ligand and the central ion [22]. Analysis of the literature shows the influence of the type of imine bridge and substituents in the phenolate ligand and solvent groups on the mechanism of the electrode process. Kinetic studies also show the influence of these parameters and the type of supporting electrolyte on the transport of charge through polymer films [3,23,25,29].

**Scheme 1:**
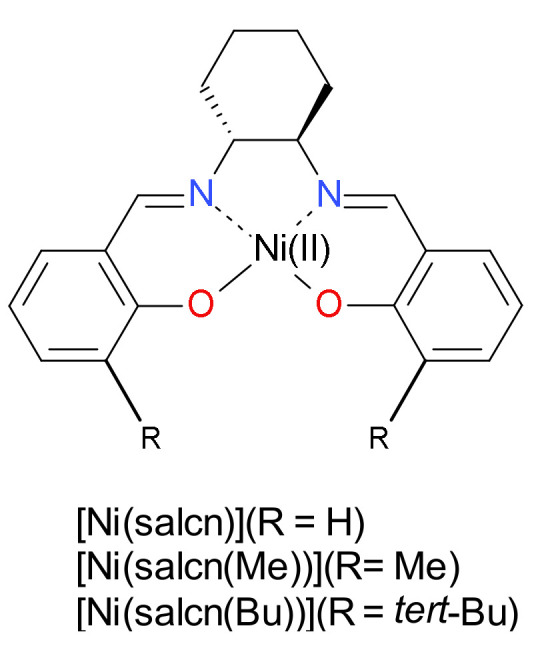
Structural formula of complexes.

Our research focuses on electrodes modified with the (±)-*trans*-*N*,*N′*-bis(salicylidene)-1,2-cyclohexanediaminenickel(II) complex ([Ni(salcn)]) and its *tert-*Bu ([Ni(salcn(Bu))]) and Me derivatives ([Ni(salcn(Me))]), substituted at the 3,3′-positions of the phenolate moieties (Scheme 1). To date, we have investigated the mechanism of complex oxidation in MeCN, CH_2_Cl_2_ and DMSO [35], and the electropolymerization mechanism of the complexes and their electroactivity [36,37]. It was found that in DMSO, the central ion is oxidized in these complexes [35]. On the other hand, in non-coordinating solvents, in complexes containing *tert*-Bu substituents and in the complex without substituents, the ligand is oxidized to the phenoxyl radical, bis phenoxyl radical and phenoxonium cation [35,36,38]. However, in the complex with Me substituents, both the ligand and the central ion are oxidized [38]. As a result of these processes, and the subsequent polymerization reaction, *phenyl-phenyl* electroactive polymer films are obtained on the surface of the electrodes. They are conductive polymers in which charge transport takes place by π electron delocalization [36].

The present paper presents the effects of the type of supporting electrolyte and solvent on the kinetics of redox processes in *poly*[Ni(salcn)], *poly*[Ni(salcn(Me))] and *poly*[Ni(salcn(Bu))] films, derived from the above-mentioned complexes. FTIR ATR and EQCM methods were used for our investigations.

## 2. Results and Discussion

### 2.1. FTIR ATR of Polymer Films

**Figure 1:**
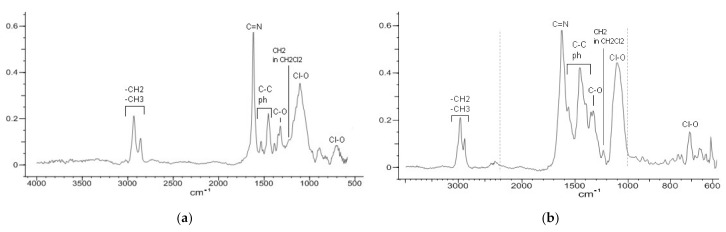
FTIR ATR spectra of *poly*[Ni(salcn)] electrosynthesized in TBAClO_4_/CH_2_Cl_2_ (*v* = 0.05 V·s^−1^, 10 cycles, vs. Ag/AgCl), (**a**) neutral, (**b**) oxidized at the anode peak potential, *E_pa_* = 0.8 V.

FTIR ATR studies of electrodes modified with polymer films, neutral and oxidized, were carried out in order to determine the type of ions involved in charge transport during electrode processes. The analysis was performed on the basis of *poly*[Ni(salcn)] synthesized and investigated in TBAClO_4_. The most characteristic bands of the neutral film (Figure 1a) were the bands at 2922 and 2881 cm^−1^, corresponding to the valence vibrations of the -CH_2_ and -CH_3_ groups of the cyclohexane groups of the polymer and the cations of the supporting electrolyte present in the film structures. Another characteristic was the band at 1624(s) cm^−1^ corresponding to the valence vibrations of the imine groups, the bands at 1536(w), 1511(w) and 1475(m) cm^−1^ corresponding to the C-C phenyl ring in plane vibration, a band at 1322(m) cm^−1^ corresponding to *υ* C-O, a band at 1236(w) cm^−1^ derived from CH_2_ out of plane bendings in CH_2_Cl_2_, and wide bands in the ranges 1220–1000(s) and 780–600(w) cm^−1^ with maxima at 1092(s) cm^−1^ and at 702(w) cm^−1^, corresponding to the vibration of the Cl-O groups in ClO_4_^−^. The presence of bands corresponding to the C-C phenyl ring in plane vibration confirmed the presence of these groups in the structure of the investigated film. This was particularly important due to the lack of bands above 3000 cm^−1^, which could be attributed to -CH stretching vibration in arenes. Most likely, these bands were superimposed on the CH_2_ and -CH_3_ bands of the cyclohexane groups of the polymer and the electrolyte cations. This explanation was confirmed by the location of the most characteristic and most intense band, corresponding to the valence vibrations of the imine groups. It occurred at slightly lower frequencies than those most frequently reported in the literature [39]. Due to the shift of the spectral bands, the band derived from CH_2_ in CH_2_Cl_2_ occurred at slightly lower frequencies than in the pure solvent [39].

In the oxidized film (Figure 1b), bands derived from *υ* CH_2_ and CH_3_ (2922(m) and 2881(m) cm^−1^), *υ* C=N (1624(s) cm^−1^) and *δ* CH_2_Cl_2_ (1236(w) cm^−1^) did not change their intensity. The same intensity of the bands at 2922(m) and 2881(m) cm^−1^ indicated that the concentration of cations in the film after the anode process did not decrease, which means that they were not involved in charge transport to ensure electroneutrality. On the other hand, the unchanged intensity of the band originating from the valence vibrations of the imine groups was an effect resulting from the location of these groups outside the coupling area. Earlier studies have shown that delocalisation occurs through C-O and Ni (II) groups as a bridge connecting phenolate groups [36]. The remaining bands increased their intensity. The increase in the intensity of the bands from the C-C phenyl ring in plane vibration (1536(w), 1511(w) and 1475(m) cm^−1^) was related to the change in the surroundings of the aromatic ring, resulting from the charge delocalisation [40]. Such changes have been shown previously in the literature [24,41]. During the anode process, due to delocalisation, more quinoid connections were formed, as a result of which an increase in the intensity of the bands or a change in their position was observed. In the presented spectrum (Figure 1b) the band was shifted from 1536(m) to 1566(s) cm^−1^. On the other hand, the increase in the band intensity at 1322(m) cm^−1^, corresponding to the C-O valence vibrations, resulted from the participation of these groups in the coupling. This delocalisation route has also been demonstrated in other salen-type complexes [24]. The bands corresponding to the vibrations of the Cl-O groups in ClO_4_^−^ (1220–1000(s) and 780–600(w) cm^−1^) also increased significantly, indicating a higher concentration of these ions in the oxidized film than in the neutral film. This result showed that the electroneutrality condition during the anode process was achieved by transporting the anions into the polymer film. Analysis of the literature shows analogous charge transport mechanisms during the oxidation of polymer films of salen-type complexes [25,26,28]. However, a slightly reduced intensity of the band derived from CH_2_ out of plane bendings in CH_2_Cl_2_ (1236(w) cm^−1^) indicated a reduced solvent content in the oxidized polymer films compared to neutral films.

In the case of electrodes modified with films derived from the [Ni(salcn(Me))] and [Ni(salcn(Bu))] complexes, the spectra differed from the spectra of the *poly*[Ni(salcn)] modified electrodes in the intensity of the bands corresponding to the CH_2_ vibration out of plane bendings in CH_2_Cl_2_. The results were exemplified by the FTIR ATR spectrum of a modified by *poly*[Ni(salcn(Me))] film electrode, synthesized and studied in TBABF_4_. The solvent band occurred at 1228(w) cm^−1^ (Figure S1, ESI). Upon oxidation of the polymer film, this band increased, indicating diffusion of the solvent into the structures of the polymer film. Another effect of the solvent compared to *poly*[Ni(salcn)] films may be related to the presence of the substituents in monomers resulting in the polymerization towards only one active position of the phenolate moieties (5,5′). After the oxidation of *poly*[Ni(salcn(Me))] and *poly*[Ni(salcn(Bu))] films, the band derived from anion vibrations, BF_4_^−^, was also increased, occurring within the range of 1150–950(m) cm^−1^, with a maximum at 1044(m) cm^−1^ (Figure S1, ESI). With the intensity of the bands corresponding to the vibrations of the -CH_2_ and -CH_3_ groups unchanged (Figure S1, bands at 2935(m) and 2859(m) cm^−1^, ESI) in the oxidized films, the increased band originating from BF_4_^−^ indicated the sole contribution of the anion in realizing the electroneutrality condition during the anode process.

### 2.2. EQCM Research

#### 2.2.1. Voltammetric Curves

**Figure 2:**
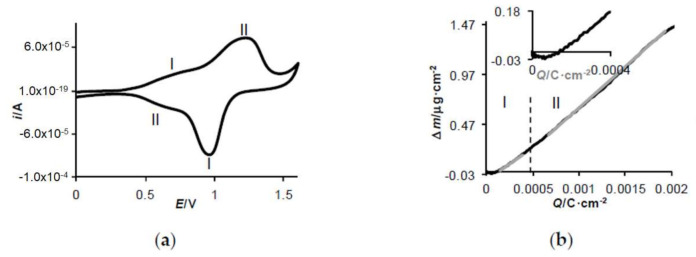
*Poly*[Ni(salcn)] (1 electropolymerization cycle) in TBAClO_4_/CH_2_Cl_2_, Pt/quarzglas, *v* = 0.05 V⋅s^−1^, vs. Ag/AgCl; (**a**) cyclic voltammogram; (**b**) plots Δ*m* vs. *Q* for anodic process; grey parts of the curve—trend lines for the Δ*m* = f(*Q*) relationship. I and II—peaks labels.

**Figure 3:**
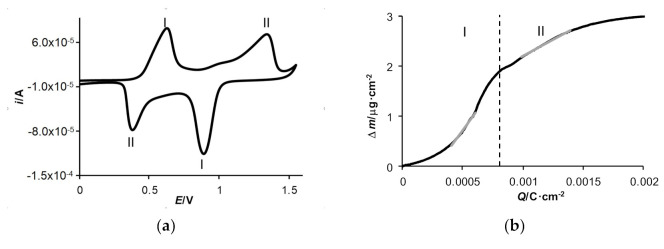
*Poly*[Ni(salcn(Me))] (1 electropolymerization cycle) in TBAClO_4_/CH_2_Cl_2_, Pt/quarzglas, *v* = 0.05 V⋅s^−1^, vs. Ag/AgCl; (**a**) cyclic voltammogram; (**b**) plot Δ*m* vs. *Q* for anodic process; grey parts of the curve—trend lines for the Δ*m* = f(*Q*) relationship. I and II—peaks labels.

**Figure 4:**
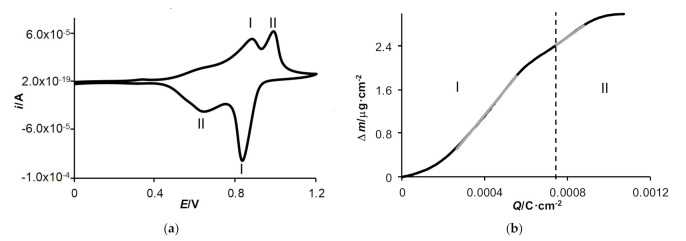
*Poly*[Ni(salcn(Bu))] (1 electropolymerization cycle) in TBAClO_4_/CH_2_Cl_2_, Pt/quarzglas, *v* = 0.05 V⋅s^−1^, vs. Ag/AgCl; (**a**) cyclic voltammogram; (**b**) plot Δ*m* vs. *Q* for anodic process; grey parts of the curve—trend lines for the Δ*m* = f(*Q*) relationship. I and II—peaks labels.

The voltammetric curves of the investigated polymers obtained, as a result of one electropolymerization cycle in the solutions of all basic electrolytes, were characterized by two oxidation peaks (Figure 2a, Figure 3a and Figure 4a and Figures S2A–S7A, ESI). As previously shown, for *poly*[Ni(salcn)] and *poly*[Ni(salcn(Bu))], they derived from the oxidation of phenolate moieties to phenoxyl radicals and bis phenoxyl radicals [38]. In the case of *poly*[Ni(salcn(Me))], they also included the oxidation of Ni (II) to Ni (III) [38]. On the other hand, in *poly*[Ni(salcn(Bu))] films, at the lowest potentials there was an additional anode signal (Figure 4a and Figure S6A, ESI), probably derived from a different geometric structure of the polymer chains, caused by the large steric effect of the substituents.

The different shape of the peaks of the studied polymers indicated that their electronic structure was sensitive to the surroundings in the phenolate moiety, which was also found in the case of other salen-type complexes [25,27]. The shape of the peaks and their potentials are also influenced by the type of supporting electrolyte. In TBABF_4_, a different nature of the curves was observed for each polymer than that recorded in the other two types of electrolytes (Figures S3A, S5A and S7A, ESI). However, the FTIR ATR studies of the oxidized film, showing an increase in the intensity of the C-C phenyl ring in plane vibration bands in relation to the neutral film, confirmed that in the TBABF_4_ solution, similarly to the others, oxidation of phenolate ligand groups took place.

#### 2.2.2. Gravimetric Curves

Gravimetric curves were obtained on the basis of the Sauerbrey equation [42]. Literature studies show that salen-complex-type films are properly rigidly bound to the surface, which allowed the authors to apply a gravimetric interpretation based on the Sauerbrey equation [25,26]. Assuming that the investigated films are rigid or not sufficiently rigid, but viscoelasticity is not their dominant feature, the application of the Sauerbrey equation is justified [26,43].

The molar masses (M) of species exchanged with the polymer film during the oxidation processes were determined based on the slopes of the linear sections of the Δ*m* = f(*Q*) relationship, for the first and second step of the anode process, from the foot of a given anode peak to the value corresponding to its development. In the case of significant non-linearity in this part, the slopes were determined from a small area around the peak potential.

Dependencies Δ*m =* f(*Q*) for *poly*[Ni(salcn)], on the basis of the processes recorded at *v* = 0.05 V·s^−1^ (Figure 2b and Figures S2B and S3B, ESI) in the applied potential range, showed slight deviations from linearity in the end sections of curves and decreasing character in the initial sections of curves 

The decreasing nature of the curves Δ*m =* f(*Q*) in the initial sections of the curves may be related to at least three mechanisms of charge transport through the polymer film [44].

**Figure 5:**
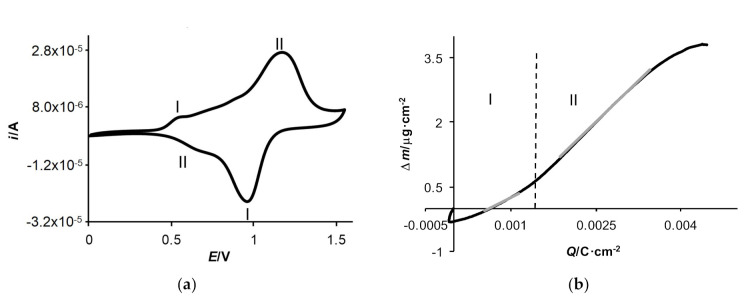
*Poly*[Ni(salcn)] (1 electropolymerization cycle) in TBAClO_4_/CH_2_Cl_2_, Pt/quarzglas, *v* = 0.01 V⋅s^−1^, vs. Ag/AgCl; (**a**) cyclic voltammogram; (**b**) plot Δ*m* vs. *Q* for anodic process; grey parts of the curve—trend lines for the Δ*m* = f(*Q*) relationship. I and II—peaks labels.

**Figure 6:**
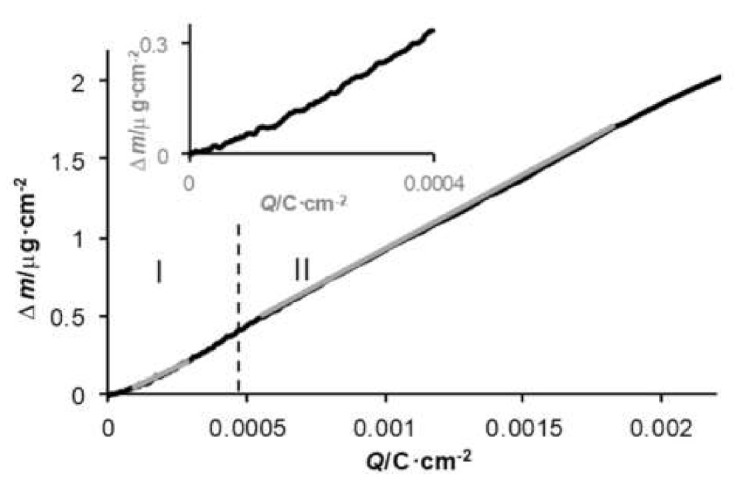
Plots Δ*m* vs. *Q* for anodic process for of *poly*[Ni(salcn)] (1 electropolymerization cycle) in TBAClO_4_/CH_2_Cl_2_, Pt/quarzglas, *v* = 0.05 V⋅s^−1^, after reduction 40 s at 0 V, vs. Ag/AgCl; grey parts of the curve—trend lines for the Δ*m* = f(*Q*) relationship.

Analysis of these possibilities requires connecting them with the values of the obtained molar masses of species exchanged with the film during the oxidation process and with the results of FTIR ATR. The IR spectra showed an increase in the anion content of the oxidized film structures, which means that anions were involved in charge transport. The analysis of the literature also points to such a mechanism as dominant for salen-type-complex polymers [25,26,28]. However, the slopes of the linear parts of the Δ*m* = f(*Q*) relationships for the first step of the oxidation process for *poly*[Ni(salcn)], in solutions of all supporting electrolytes, did not correspond to the *M* values for pure anions resulting from Faraday’s laws. The calculated *M* values (Table 1) were lower than expected for individual anions (145 g·mol^−1^, 99.5 g·mol^−1^ and 87 g·mol^−1^ for PF_6_^−^, ClO_4_^−^ and BF_4_^−^, respectively) and they were 0.5–0.7 of the *M* values. Such a result could indicate diffusion of the solvent from the polymer film during the first step of the oxidation process [44,45]. However, the curves Δ*m* = f(*Q*) obtained during the anode processes at scan rate *v* = 0.05 V·s^−1^ were not monotonic; in the initial sections of the curves the mass decreased (Figure 2b and Figures S2B and S3B, ESI). The charge transport mechanism, based on the share of pure anion and solvent, should show a rectilinear relationship Δ*m* = f(*Q*) in the whole range [44,45]. Other possibilities to consider for the decreasing mass at the beginning of the curves shown in Figure 2b and Figures S2B and S3B, ESI, are the mechanisms of “non-exchangeable” ions or ions association with the polymer matrix [44]. The first of these possibilities occurs because of the geometric aspects of the ions. Both this mechanism and the second can be excluded because the anions of the supporting electrolytes we used were not large and possessed poor coordinating properties, and the analysis of FTIR ATR spectra did not show a decreased share of cations in the oxidized films in relation to neutral species (Figure 1). In the case of an anion association, the relationship Δ*m* = f(*Q*) is linear in the whole range [44]. The solution to the problem was provided by EQCM studies conducted at *v* = 0.01 V·s^−1^ and at *v* = 0.05 V·s^−1^, but recorded after a longer reduction (40 s at *E* = 0 V) preceding the anodic process in the applied potential range. The curves Δ*m* = f(*Q*) (Figure 5b and Figures S8 and S9, ESI), obtained based on processes recorded for scan rate *v* = 0.01 V·s^−1^, showed an even greater reduction of the masses in the initial sections of the curves than the curves in Figure 2b and Figures S2B and S3B. There was also a decrease in the charge in this part of the curves (Figure 5b and Figures S8 and S9, ESI). The simultaneous decrease in these two values indicated that the film was not fully neutral and that, at the beginning of the anodic process carried out at the lowest potentials, a reduction takes place. Hence, the reduction in film mass was due to anions leaving the film. The confirmation of the hypothesis for the process carried out at *v* = 0.01 V·s^−1^ were the results obtained for the process carried out at *v* = 0.05 V·s^−1^ in the applied potential range preceded by a reduction lasting 40 s, at a potential of 0 V. The relationships Δ*m* = f(*Q*) (Figure 6 and Figures S10 and S11, ESI) in the initial sections of the curves did not show a decreasing character, and the molar masses of species participating in the oxidation process in step I, determined on the basis of the linear parts of these relationships, were higher, except for *M* in TBABF_4_. Taking into account the above considerations, the FTIR ATR spectra, and the fact that the obtained *M* values for the first step of the oxidation process occurring in *poly*[Ni(salcn)] were lower than those resulting from Faraday’s laws for pure ions, it can be concluded that charge transport occurred through the penetration of anions into the polymer film and the diffusion of the solvent in the reverse direction. Such a mechanism is justified for *poly*[Ni(salcn)] films, most probably due to electropolymerization in the directions of both active positions of phenolate groups (*ortho*- and *para*-). The removal of the solvent accompanying the penetration of the anions was therefore intended to obtain the volume required for these anions.

The second step of the oxidation processes in *poly*[Ni(salcn)] was characterized by slopes of the linear parts of the curves Δ*m* = f(*Q*), indicating the dependence of the charge transport mechanism on the type of the primary electrolyte (Table 1, 0.05 V·s^−1^ after a reduction lasting 40 s). In the TBAClO_4_ solution, the value of *M* exactly corresponded to that of the pure anion according to Faraday’s laws. In the TBABF_4_ solution, the *M* value was lower than expected for the BF_4_^−^ ion. On the other hand, in the TBAPF_6_ solution, the *M* value was higher than expected for the PF_6_^−^ ion and indicated that the transport of the anion into the polymer film was accompanied by the transport of the solvent in the same direction [25,26]. 

Analyzing the molar masses for each supporting electrolyte, they were higher for the second step of polymer oxidation than for the first step (Table 1). Regardless of the step, the *M* values were the lowest in the BF_4_^−^ solution. 

In the case of the other polymers, (*poly*[Ni(salcn(Me))] and *poly*[Ni(salcn(Bu))]), for both scan rates (*v* = 0.01 and 0.05 V·s^−1^), the relationships Δ*m* = f(*Q*) did not show mass reduction in the initial sections of the curves (Figure 3b, Figure 4b and Figures S4B, S5B, S6B and S7B, ESI) and the calculated *M* values on the basis of linear parts of the Δ*m =* f(*Q*) relationship (Table 2) were higher than expected for pure anions (except for the *M* value obtained for the second step of the *poly*[Ni(salcn(Me))] process in TBABF_4_). In light of the analysis carried out for *poly*[Ni(salcn)] films, this indicated a charge transport mechanism based on the penetration of anions into the polymer film, accompanied by solvent diffusion in the same direction. FTIR ATR studies showed an increased proportion of both anions and solvent in the oxidized films (Figure S1, ESI). Molar mass values were comparable for both scan rates, which resulted in the independence of the charge transport mechanism from *v*. The molar masses for both *poly*[Ni(salcn(Me))] and *poly*[Ni(salcn(Bu))] were greater in the first step of the electrode process than in the second step (Table 2), unlike that observed for *poly*[Ni(salcn)] (Table 1). 

The dependence of M on the type of electrolyte was more visible in the case of *poly*[Ni(salcn(Me))], especially at the rate *v* = 0.01 V·s^−1^ (Table 2). More moles of the solvent penetrated the *poly*[Ni(salcn(Bu))] films than *poly*[Ni(salcn(Me))] ones; in the case of *poly*[Ni(salcn(Bu))], it was the same in all electrolyte solutions, except for the second oxidation step in TBABF_4_. This may have been due to the different structural properties of the polymers resulting, inter alia, from the different steric effects of substituents of different sizes.

When comparing the M values in solutions of different electrolytes, the smallest number of moles of solvent was also transported in the TBABF_4_ medium; in the second step even diffusion of the solvent from the polymer film was observed. The relationship of the number of moles of diffusing solvent on the type of electrolyte may be related to the size of anions. The BF_4_^−^ ion was the smallest among the investigated anions (ion diameter BF_4_^−^ = 0.334 nm [46]). The other ions, in the presence of which mass transport takes place to a greater extent, had larger sizes (ion diameters 0.370 and 0.435 nm for ClO_4_^−^ and PF_6_^−^, respectively [46]).

The dependence of the charge transport on the type of solvent was demonstrated in the case of *poly*[Ni(salcn)].

**Figure 7:**
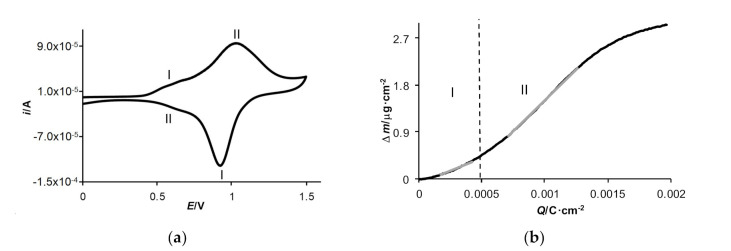
*Poly*[Ni(salcn)] (1 electropolymerization cycle) in TBAClO_4_/MeCN, Pt/quarzglas, *v* = 0.05 V⋅s^−1^, vs. Ag/AgCl; (**a**) cyclic voltammogram; (**b**) plot Δ*m* vs. *Q* for anodic process; grey parts of the curve—trend lines for the Δ*m* = f(*Q*) relationship. I and II—peaks labels.

In MeCN, the molar masses of the first step of the process were as expected for pure anions (Table 1 based on Figure 7b and Figures S12 and S13B, ESI). However, in the second step of the process conducted in the MeCN, the solvent was transported along with the ions. For each type of supporting electrolyte, a much larger number of moles of solvent penetrated into the film structures than in the CH_2_Cl_2_ process (Table 1). This result was most likely related to the lower MeCN viscosity (MeCN = 0.316, CH_2_Cl_2_ = 0.43 mP·s), which facilitated the movement of ions and solvent.

### 2.3. Cyclic Voltammetry

#### 2.3.1. Electroactive Surface Coverage

**Figure 8:**
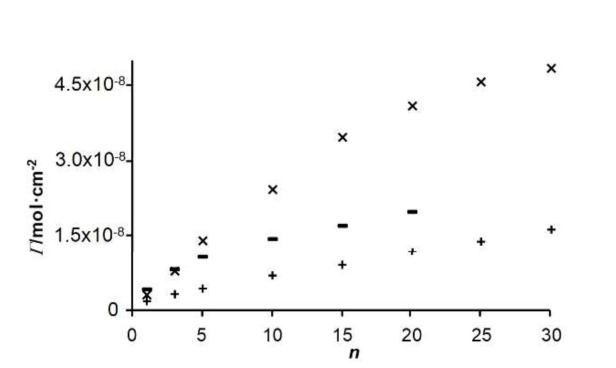
Plots of the apparent surface coverage (*Γ*) vs. the number of electropolymerization cycles (*n*) for: (×) *poly*[Ni(salcnMe)], (−) *poly*[Ni(salcn)], (+) *poly*[Ni(salcn(Bu))].

The electroactive surface coverage was determined using Faraday’s law *Γ* = *Q*/*zFA* [47]. The relations *Γ* = f(*n*) (*n*—number of electropolymerization cycles), for all polymers and in solutions of all supporting electrolytes, showed an increasing characteristic (Figure 8). However, only for *poly*[Ni(salcn(Bu))] was this relationship linear over the whole range of electropolymerization cycles, which indicated that the conductivity of the film did not limit the electrolytic deposition under the investigated conditions. Such features of the polymer have also been noticed in the literature for another salen-type complex [23]. In the case of films of the other polymers, deviations from linearity were observed, the highest for *poly*[Ni(salcn)]. The reason was most likely the structure of this polymer, formed as a result of electropolymerization towards both active positions of the phenolate groups [36], which may have impeded conductivity in the thicker films. In the case of the *poly*[Ni(salcn(Me))] polymer, most probably due to the presence of substituents on the phenolate moieties, deviations from linearity occurred with thicker films. A decrease in electroactive surface coverage with an increase in film thickness was observed in the literature for other conductive polymers, such as polypyrrole, porphyrin complexes [48] and salen-type complexes [17,27].

Comparing the thin films of the investigated polymers, obtained as a result of one to three cycles, it can be seen that the *poly*[Ni(salcn)] films were characterized by the highest surface coverage. On the other hand, the lowest values of *Γ* occurred for *poly*[Ni(salcn(Bu))] films, despite the lack of limitations in electrodeposition of the film. The reason seemed to be less efficient electropolymerization [36], due to the large steric effect of the substituents.

#### 2.3.2. Kinetic Analysis

The nature of the voltammograms of the investigated polymers depended on the film thickness, the polarization rate of the electrode, and the type of supporting electrolyte and solvent.

**Figure 9:**
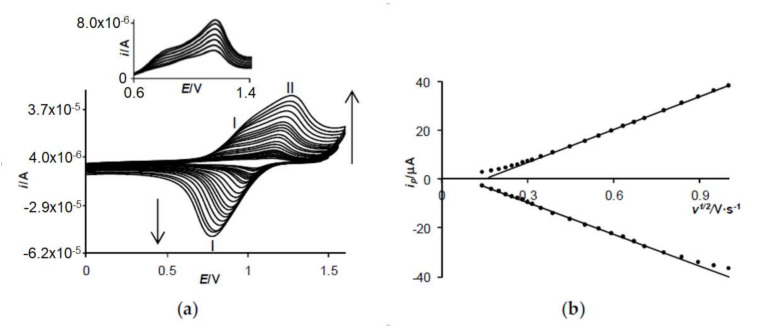
*Poly*[Ni(salcn)] (3 electropolymerization cycles) in TBAClO_4_/CH_2_Cl_2_, Pt, vs. Ag/AgCl; (**a**) the lower part—cyclic voltammograms for *v* = 0.02–1 V⋅s^−1^, the upper part—anodic voltammograms for *v* = 0.02–0.08 V⋅s^−1^; (**b**) plots *i_p_* vs. *v*^1/2^ for oxidation and reduction processes. I and II—peaks labels.

In case of *poly*[Ni(salcn)] in CH_2_Cl_2_, for its thin films, obtained by up to three electropolymerization cycles in TBAClO_4_ solution, two-step electrode processes were observed at lower scan rates (Figure 9a). At higher scan rates, the first step of the electrode process was noticed only in the form of an inflection on the signal of the second step of the process Figure 9a). For thicker films, with higher scan rates, the process was only one-step (Figure S14A, ESI). In contrast, in the TBABF_4_ solution, in CH_2_Cl_2_, at higher scan rates, only one step of the electrode process was observed (Figure S15A, ESI). This variability in the nature of the voltammograms indicated kinetic limitations that proceeded with the thickness of the films.

**Figure 10:**
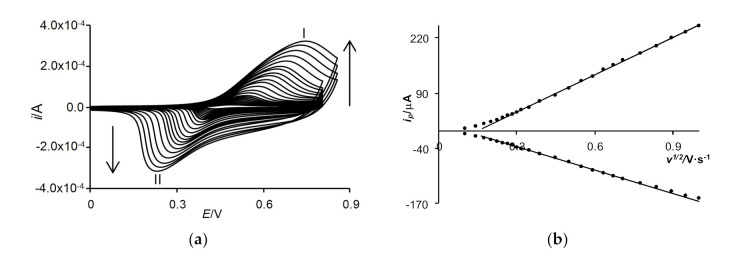
*Poly*[Ni(salcn(Me))] (15 electropolymerization cycles) in TBAClO_4_/CH_2_Cl_2_, Pt, *v* = 0.01–1 V⋅s^−1^, vs. Ag/AgCl; (**a**) cyclic voltammograms for one-step process, peak notations were used adequate to peak designation in a two-step process, see Figure 3; (**b**) plots *i_p_* vs. *v*^1/2^ for oxidation and reduction processes. I and II—peaks labels.

**Figure 11:**
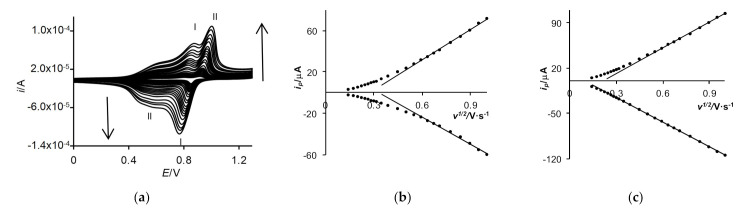
*Poly*[Ni(salcn(Bu))] (10 electropolymerization cycles) in TBAClO_4_/CH_2_Cl_2_, Pt, *v* = 0.02–1 V⋅s^−1^, vs. Ag/AgCl; (**a**) cyclic voltammograms; (**b**) plots *i_p_* vs. *v*^1/2^ for 1st oxidation step of the process and 2nd reduction step*; (***c**) plots *i_p_* vs. *v*^1/2^ for 2nd oxidation step of the process and 1st reduction step. I and II—peaks labels.

Kinetic limitations were also observed for the *poly*[Ni(salcn(Me))] polymers. Thicker films, in the second step, at low rates, were oxidized to a very small extent (Figure S16, ESI), and the first step of the electrode process became more and more electrochemically irreversible with increasing scan rate, both in TBAClO_4_ and TBABF_4_ (Figure 10a and Figure S17A, ESI). On the other hand, kinetic limitations were least visible in the voltammograms obtained for *poly*[Ni(salcn(Bu))]. Regardless of the film thickness, they oxidized in two steps, and the anode and cathode peak potentials shifted less (Figure 11a and Figure S18A, ESI) than was observed on voltammograms for *poly*[Ni(salcn(Me))] (Figure 10a and Figure S17A, ESI). The exception was the TBABF_4_ medium—at the highest scan rate the second anodic peak for *poly*[Ni(salcn(Bu))] disappeared (Figure S19A, ESI). 

The electrode processes in the films of all polymers were not electrochemically reversible. The greatest irreversibility was observed in the TBABF_4_ solutions. The potential differences of the anode and cathode peaks for the *poly*[Ni(salcn(Me))] film were greater in TBABF_4_ (Figure S17A, ESI) than in TBAClO_4_ (Figure 10a). In contrast, in the voltammograms of the films of *poly*[Ni(salcn)] (Figure S15A, ESI) and *poly*[Ni(salcn(Bu))] (Figure S19A, ESI) in TBABF_4_, the reduction peaks were absent or hardly visible. Despite significant electrochemical irreversibility, the polymer films were stable during the electrode processes. The successively recorded voltammetric curves did not show any significant differences (Figures S20 and S21, ESI).

In MeCN, the voltammetric curves were less electrochemically irreversible (Figures S22A and S23A, ESI) than the curves recorded in CH_2_Cl_2_ (Figure 9a and Figure S15A, ESI), which was also shown on the basis of previous electrocatalytic studies [49].

The analysis of the kinetics of the electrode processes was carried out on the basis of *cD*^1/2^, values. For *poly*[Ni(salcn(Me))], they were determined only for the first step of the anode process and the corresponding step of the cathode process, due to the disappearance of the second step of the anode process with an increase in v. For all polymers, these parameters were determined for such film thicknesses, which to a relatively large extent showed the rectilinear relationship of the function *i_p_* = f(*v*^1/2^), and thus ensured the diffusion process. Deviations from linearity in the initial section of the relationship *i_p_* = f(*v*^1/2^) (e.g., Figure 9b) resulted from the surface process and indicated that a given film thickness at lower polarization rates of the electrode ensured oxidation of all active centres of the film ([50], p. 595). On the other hand, deviations from linearity in the final segment of the relationship *i_p_* = f(*v*^1/2^ (e.g., Figure S15B, ESI) resulted from kinetic constraints for the largest values of scan rates. They were especially visible for the polymer films of *poly*[Ni(salcn)] and *poly*[Ni(salcn(Me))].

Analyzing the values of *cD*^1/2^ presented in Table 3, Table 4 and Table 5 on the basis of Figure 9b, Figure 10b, Figure 11b,c and Figures S14B, S15B, S17–S23, S18C and S19C, ESI, it can be seen that, both in the anode and cathode processes, these values for each of the investigated polymers, obtained with the same number of cycles, were higher in the TBAClO_4_ solution than in TBABF_4_, and in each of these solutions were lower than in TBAPF_6_ ( Table 3, Table 4 and Table 5). Taking into account the different types of electrolytes used to electropolymerize a given complex, the c values in the films thus obtained (due to the same number of electropolymerization cycles) may differ, which makes it impossible to compare the *cD*^1/2^ values. Therefore, in order to determine the dependence of kinetics on anion size, electropolymerization was carried out in TBAPF_6_ solution, and redox switching of modified electrodes in solutions of all investigated electrolytes. For each polymer, the *cD*^1/2^ values in TBAPF_6_/TBAClO_4_ (electropolymerization in TBAPF_6_, redox switching in TBAClO_4_) and in TBAPF_6_/TBABF_4_ (electropolymerization in TBAPF_6_, redox switching in TBABF_4_) were higher than the values obtained in TBAPF_6_ (electropolymerization and redox switching in TBAPF_6_) (Table 3, Table 4 and Table 5). The results showed that after electropolymerization carried out in the anion solution with the largest radius, the rate of electrode processes taking place on the modified electrodes was the highest in the solution of the smallest anions.

However, the least visible effect was in the *poly*[Ni(salcn)] films, both on the basis of *cD_a_*^1/2^ and *cD_c_*^1/2^ values, most likely due to electropolymerization towards both active positions of the phenolate moieties in the monomers. The *cD_a_*^1/2^ values in TBAPF_6_/TBAClO_4_ were only about 1.2 times higher than those obtained in TBAPF_6_ (Table 3). A slightly greater difference was obtained in TBAPF_6_/TBABF_4_ versus TBAPF_6_, most likely due to the greater difference between the anion sizes used for electropolymerization and redox switching. In the case of the other polymers, these differences were greater (Table 4 and Table 5). For *poly*[Ni(salcn)], both *cD_a_*^1/2^ as well as *cD_c_*^1/2^ values increased with increase in the number of electropolymerization cycles in each electrolyte solution, but to a lesser extent, indicating progressive kinetic limitations. For films obtained as a result of recording 15 and 20 cycles, values were even lower than for films obtained as the result of recording 10 cycles. 

There were greater kinetic constraints during the cathode process than during the oxidation process. At low electrode polarization rates, 0.01–0.06 V·s^−1^, a surface process was observed in the TBAClO_4_ solution during the anodic process (Figure 9b), (the deviation from the linearity of the *i_pa_* = f(*v*^1/2^) relationship, in the initial section), which did not not occur during the cathodic process. In addition, the *cD_c_*^1/2^ values were lower than the corresponding *cD_a_*^1/2^ values (Table 3), although, from the *cD_c_*^1/2^ < *cD_a_*^1/2^ relationship, it was difficult to unequivocally conclude that *D_c_* < *D_a_*, due to the electrochemical irreversibility of the electrode process, which means that the concentration of the active centres undergoing reduction may have been lower than those undergoing the previous oxidation reaction. However, EQCM studies showed that, in the case of this polymer, there was practically no solvent transport into the film structures during the oxidation processes (Table 1). Hence, it can be assumed that the concentrations of the active sites after oxidation will not have changed significantly and, from the *cD_c_*^1/2^ < *cD_a_*^1/2^ relationship, it was possible to conclude the *D_c_* < *D_a_* relationship.

The slower rate of reduction processes than oxidation processes in *poly*[Ni(salcn)] films was the opposite effect to that described in the literature for other salen-type complex polymers [23,25]. The explanation for our results seems to be the structure of the cross-linked *poly*[Ni(salcn)] polymer, which, during the transport of anions into the film by forced oxidation process, may have had limited scope to increase its volume. Hence, in the first step of the oxidation process, diffusion of the solvent from the films was observed in order to make room for the anions. The mass of such a film, increased during the oxidation process, undoubtedly reduced the free space of the film and may have hindered the transport of anions into the solution during the reduction process.

The *cD_a_*^1/2^ values obtained for the films of the other two polymers, *poly*[Ni(salcn(Me))] and *poly*[Ni(salcn(Bu))] in TBAPF_6_/TBAClO_4_ and TBAPF_6_/TBABF_4_, were similar to those for the films of *poly*[Ni(salcn)], indicating that the transport of charge through the polymer film was determined by the transport of anions and not the movement of electrons through the conductive backbone of the polymers (Table 4 and Table 5). After electropolymerization in the TBAPF_6_ solution, the highest values were obtained in the solution of the smallest anion, and the lowest in the largest anion solution, PF_6_^−^ (Table 4 and Table 5), which indicated that the optimal solution for increasing the speed of electrode processes was the greatest possible difference between the anions used for electropolymerization and redox switching of the modified electrodes.

The deviations from linearity in the initial sections of the relation *i_pa_* = f(*v*^1/2^), both for anode and cathode processes (Figure 10b and Figure S17B, ESI), indicated easier kinetics in *poly*[Ni(salcn(Me))] than *poly*[Ni(salcn)] films, obtained by the same procedure. The results of the EQCM studies in TBAPF_6_ and TBAClO_4_ solutions showed that, both in the first and second step of the oxidation process, the transport of anions into the films was accompanied by diffusion of the solvent in the same direction (Table 2). 

In the case of *poly*[Ni(salcn(Bu))], the type of electrolyte had the greatest impact on the kinetics of the redox processes, pointing to ion transport as a decisive step in the transport of charge through the polymer film. The lowest values of *cD_a_*^1/2^ for modified electrodes in the TBAPF_6_ solution became two and more times higher in TBAPF_6_/TBABF_4_ (Table 5), while for *poly*[Ni(salcn(Me))] films in TBAPF_6_/TBABF_4_, these values were less than two times higher than TBAPF_6_ (Table 4). Efficient anode kinetics were also evidenced by the largest deviations from linearity in the initial sections of the *i_pa_* = f(*v*^1/2^) relation, even for the thickest films (Figure 11b,c and Figure S18B,C, ESI), and no deviations from linearity in the final sections of these relationships were observed. Only in TBABF_4_ did such deviations occur (Figure S19B,C, ESI). The reason for the fastest kinetics of electrode processes in *poly*[Ni(salcn(Bu))] films, and the apparent influence of the type of ion on these kinetics, was probably the structure of the monomer. Furthermore, only in the case of this polymer in TBAPF_6_/TBAClO_4_ was there a *cD_cI_*^1/2^ > *cD_aII_*^1/2^ relationship (Table 5), analogous to that described in the literature for electrodes modified with salen-type complexes [23,25], which showed the relationship *D_cI_* > *D_aII_*. The reason for the higher *D_cI_* values than *D_aII_* may have been the larger film volume obtained during oxidation, which facilitated the removal of ions and solvent from the film during the reduction process. The EQCM results showed that, in the case of this polymer, the greatest number of moles of the solvent penetrated into the structure of its films during oxidation processes four moles per one mole of anions in the first step of the process, about two moles in the second step (Table 2)). 

Comparing the *cD_c_*^1/2^ values obtained in the TBAClO_4_ solution (Table 5), it can be seen that *cD_c1I_*^1/2^ < *cD_cI_*^1/2^, which may indicate the effect of the reduced film volume, after the first step of the reduction process, as a result of removing ions and solvent from the film, on hindering charge transport in the next reduction step. 

On the other hand, when comparing the values of *cD_a_*^1/2^ (Table 5), an inverse relationship, *cD_a1I_*^1/2^ > *cD_aI_*^1/2^, in each electrolyte solution was observed. Taking into account the increase in the volume of films during the successive steps of the oxidation process, the dependence *cD_a1I_*^1/2^ > *cD_aI_*^1/2^ clearly showed the *D_a1I_* > *D_aI_* relationship. Here, the effect of reorganization of the film structure after the first step of the electrode process ([50], p. 505) was most likely reflected, as a result of the interaction of functional groups ([50], p. 505). Consequently, the film adopted a structure that was more conducive to charge delocalization ([50], p. 505). This process was facilitated by the increased volume of the film after the first step of the oxidation process. 

Voltammetric investigations in MeCN solutions allowed assessment of the influence of the solvent on the kinetics of the electrode processes. In MeCN, the *cD_a_*^1/2^ values for *poly*[Ni(salcn)] polymer films were about 1.3 times higher than in CH_2_Cl_2_, relative to the electrolytes of the same type (Table 3). Moreover, for any of the tested film thicknesses, no decrease in the *cD_a_*^1/2^ value was observed with increase in film thickness. The relationship *i_p_ =* f(*v*^1/2^) (Figures S22B and S23B,C, ESI) did not substantially deviate from linearity in the end sections of these functions (but with slight deviations in TBABF_4_). EQCM studies showed that the molar masses of the species transported in the films were also greater in MeCN than in CH_2_Cl_2_ (Table 1). Moreover, for any of the steps in the oxidation process, no diffusion of the solvent from the polymer films was observed in order to make room for the electrolyte ions transported into the films. These features indicated easier charge transport in MeCN than in CH_2_Cl_2_. The reason for faster kinetics in MeCN than in CH_2_Cl_2_ may have been the lower viscosity of MeCN, facilitating diffusion. Another reason may have been that the MeCN donor number (DN) was higher. This value for MeCN was 14.1, while for CH_2_Cl_2_ it was 0. The consequence of a higher value of DN was an anion association which increased the anion radii and thus larger gaps in the polymer films obtained after the electropolymerization process, which may have had a positive effect on the conductive backbone, facilitating the relocation of the charge.

## 3. Materials and Methods

### 3.1. Materials

Ligands: (±)-*trans*-*N,N*′-bis(salicylidene)-1,2-cyclohexanediamine (salcn), (±)-*trans*-*N,N′*-bis(3-methylsalicylidene)-1,2-cyclohexanediamine (salcn(Me)) and (±)-*trans*-*N,N*′-bis(3-*tert*-butylsalicylidene)-1,2-cyclohexanediamine (salcn(Bu)) were synthesized in the stoichiometric reaction of *trans*-1,2-diaminocyclohexane and salicylic aldehyde in 96% ethanol solution according to [51]. Elemental analyses, ^1^H-NMR, IR and UV-VIS results, confirming the obtained compounds are presented in [35,36].

Complexes of the investigated ligands with Ni(II) ions: [Ni(salcn)], [Ni(salcn(Me))] and [Ni(salcn(Bu))] were obtained according to [52]. Elemental analyses, IR, UV-VIS, magnetic susceptibility and ^1^H-NMR (only for [Ni (salcn (Me))]) results confirming the obtained complexes were previously published [35,36]. Solutions of the complexes were prepared and deoxidized with argon directly before measurement.

Tetrabutylammonium perchlorate (TBAClO_4_), tetrabutylammonium tetrafluoroborate (TBABF_4_), and tetrabutylammonium hexafluorophosphate (TBAPF_6_) were obtained from Fluka. Potassium hexacyanoferrate(III) was of analytical grade, from POCH. Acetonitrile (MeCN) and methylene chloride (CH_2_Cl_2_) were of HPLC grade, from Baker. All of the chemicals were used as received.

### 3.2. Instruments

Cyclic voltammetry was carried out using AUTOLAB PGSTAT 10 Eco Chemie in a three-electrode system. A platinum disk electrode (MINERAL), modified with polymer nickel complexes (Pt*poly*[Ni(salcn(R))]), was used as a working electrode. The Ag/AgCl (1 mol·dm^−3^ KCl), connected to the bulk of the solution by a Luggin capillary, was used as a reference electrode. A platinum plate was used as a counter electrode. Measurements were recorded in CH_2_Cl_2_/TBAClO_4_ and CH_2_Cl_2_/TBABF_4_ (0.1 mol·dm^−3^), deoxidized with argon. All potentials are reported relative to the Ag/AgCl (1 mol·dm^−3^ KCl) electrode. The platinum disc electrode was cleaned in HNO_3_ and aqueous suspension of 0.05μm alumina micropolish before each measurement.

EQCM measurements were performed using a module Autolab Electrochemical Quartz Crystal Microbalance for the AUTOLAB PGSTAT 302N, fitted with 6 MHz, AT-cut crystals coated with Pt. A platinum wire was used as a counter electrode and Ag/AgCl (1 mol·dm^−3^ KCl) was used as a reference electrode.

FTIR ATR spectra were performed on a Nicollet 8700 Thermo Scientific (Boston, MA, USA), on platinum disk electrodes, modified with the polymers obtained after electropolymerization process.

### 3.3. Electrode Modification Procedure

The platinum electrodes were modified by anodic electropolymerization. The process consisted in recording 1–30 cyclic voltammetric curves in solutions of complexes with a concentration of 10^−3^ mol·dm^−3^, in positive potential ranges. For the [Ni(salcn)] complex, a maximum of 20 cycles were recorded due to kinetic limitations. TBAClO_4_, TBABF_4_ and TBAPF_6_ were used as supporting electrolytes. The electropolymerization was carried out in the CH_2_Cl_2_ medium, and, in the case of [Ni(salcn)], also in acetonitrile (MeCN). The other complexes did not dissolve in MeCN. The curves were recorded in the potential ranges covering the three-step complex oxidation process [36]. The films obtained under these conditions had higher concentrations of surface centres, especially *poly*[Ni(salcn(Bu))] films [38]. As a result of this process, yellow, electroactive polymer films, well-adhered to the electrode surface, were obtained. The electrodeposition processes were carried out at the polarization rate of the working electrode *v* = 0.05 V·s^−1^. The thickness of the films was controlled by the number of electropolymerization cycles.

### 3.4. Procedures for Investigating the Modified Electrodes

Electrodes (platinum disc and quarzglas resonator) modified before electrochemical and electrochemical-gravimetric measurements were washed with a solvent and conditioned in a solvent for 10 min in order to remove as much of the complex as possible from the film structures. It was then conditioned in a supporting electrolyte solution for 5 min in order to stabilize the chemical equilibrium prior to the electrode process. For the electrodes prepared in this way, the first and the second voltammetric curve were basically the same.

The first cycles were recorded during electrochemical and electrochemical-gravimetric measurements in electrolyte solutions.

The number of electrons for the anode processes (z) was estimated according to the procedure described in [24], based on comparing the charge under the anodic part of the electropolymerization curve, obtained as a result of 1 cycle, and the charge under the anodic part of the voltammetric curve, recorded on the electrode modified in this way, in the electrolyte solution, on the basis of the relationship: *Q_po_*_l *a*_/*Q_a_* = (2 + z)/z, (*Q_pol a_*—charge under the anodic part of the electropolymerization curve; *Q_a_*—charge under the anodic part of the curve, recorded on the electrode modified in the electrolyte solution; 2—number of electrons related to complex oxidation). The curves were recorded at *v* = 0.01 V·s^−1^ in order to eliminate diffusion effects. The *z* values for *poly*[Ni(salcn)], *poly*[Ni(salcn(Me))] and *poly*[Ni(salcn(Bu))] were estimated to be 0.9, 0.8 and 0.6, respectively.

The study of the kinetics of the electrode processes taking place on modified electrodes was carried out using the cyclic voltammetry method, in TBAClO_4_ and TBABF_4_ solutions, at scan rates 0.01–1 V·s^−1^, in the potential ranges including the two-step process of oxidation of polymer films, to bisphenoxyl radicals. The third step of polymer oxidation was hardly visible [37]. Due to the lack of electrochemical reversibility of the electrode processes, the potential range was increased along with the increase in the scan rate, which facilitated the precise determination of the currents of the last anode peaks and did not significantly affect the obtained results. The kinetic analysis was carried out on the basis of the *cD*^1/2^ values, determined on the basis of the Randles–Sevcik equation for irreversible processes, with the slope of the *i_p_* = f(*v*^1/2^) relationship. The values of the transition coefficients were determined on the basis of the (1 − *α*)*n_α_* = 47.7/(*Ep_a_* − *Ep_a_*_/2_) and *αn_α_* = 47.7/(*Ep_c/2_* − *Ep_c_*) relationships, based on the voltammetric curves recorded at the electrode polarization rates for which these values were constant (*v* > 0.1 V·s^−1^). Values of (1 − *α*)*n_α_* for *poly*[Ni(salcn)], *poly*[Ni(salcn(Me))] and *poly*[Ni(salcn(Bu))] were estimated as: 0.25, 0.27 and 0.33—I step, 0.35—2nd step, respectively. On the other hand, the values of *αn_α_* for *poly*[Ni(salcn)], *poly*[Ni(salcn(Me))] and *poly*[Ni(salcn(Bu))] were estimated at: 0.26, 0.35 and 0.38—I step, 0.36—II step.

The electroactive surface coverage Γ was determined on the basis of voltammograms recorded on modified electrodes, at low scan rates *v* = 0.01 V·s^−1^, due to the achievement of conditions enabling complete oxidation of the polymer film.

The EQCM tests were carried out for modified electrodes obtained as a result of recording one electropolymerization cycle, at *v* = 0.05 V·s^−1^, in TBAPF_6_, TBAClO_4_ and TBABF_4_ solutions. The voltammetric and gravimetric curves on the modified electrodes were recorded in the potential ranges covering the two-step process of oxidation of polymer films, with the electrode polarization rates *v* = 0.01 and 0.05 V·s^−1^. The oxidation process was preceded by the reduction of films, carried out at potential *E* = 0 V for 10 s, and, in the case of *poly*[Ni(salcn)], for *v* = 0.05 V·s^−1^ also for 40 s. This ensured that inert films were obtained, and the mass and charge recorded during the anode process corresponded only to that exchanged during oxidation. The sensitivity coefficient of the quarzglas was determined based on silver electrodeposition tests.

FTIR ATR studies were carried out for modified electrodes obtained as a result of recording 10 electropolymerization cycles. Modified electrodes were washed with solvent (0.5 cm^3^) and dried at room temperature for 20 min. Spectra were recorded for neutral and oxidized films, at a potential value equal to 0.8 V. Under these oxidation conditions, EQCM studies showed that it was easiest to compare the effect of the solvent on charge transport.

## 4. Conclusions

Platinum electrodes modified with conductive polymers derived from *poly*[Ni(salcn)], *poly*[Ni(salcn(Me))] and *poly*[Ni(salcn(Bu))] complexes were obtained by anodic electropolymerization.

Charge transport through polymer films of modified electrodes was analyzed based on FTIR ATR spectroscopy, cyclic voltammetry and EQCM, in TBAPF_6_, TBAClO_4_ and TBABF_4_ solutions, in CH_2_Cl_2_ and MeCN. It was shown that in CH_2_Cl_2_, anions and a solvent were transported into *poly*[Ni(salcn(Me))] and *poly*[Ni(salcn(Bu))] films during the oxidation process. Only anions were transported into the films of the cross-linked *poly*[Ni(salcn)] polymer, while the solvent in the first step of the oxidation process diffused in the opposite direction. In this way, this polymer provided a place for anions transported into its structure.

It was found that the optimal solution for effective charge transport was to use the electrolyte with the largest anion (TBAPF_6_) for electropolymerization, and the smallest anion (TBABF_4_) for redox switching of the modified electrodes. For each type of polymer, under these conditions, the *cD*^1/2^ values were the highest.

Transport of the charge was the easiest through the films of *poly*[Ni(salcn(Bu))], most likely due to the high steric effect of the *tert*-Bu substituents. This was evidenced by the highest values of *cD*^1/2^, the largest amount of solvent diffusing into the structures of its films (*n*_s_), and the largest deviations from linearity in the initial sections of the relation *i_pa_* = f(*v*^1/2^), indicating significant participation of the surface process. The structures of *poly*[Ni(salcn(Bu))] films, regardless of their thickness and type of electrolyte, did not block the solvent movement, as indicated by the same *n_s_* values in the solutions of each of the electrolytes used, except for the second step of the electrode process in TBABF_4_.

The kinetics of the electrode processes in *poly*[Ni(salcn)] films were faster in MeCN than in CH_2_Cl_2_, most likely due to the lower viscosity of MeCN, facilitating diffusion, as evidenced by higher values of *cD*^1/2^ and *n_s_*, and smaller deviations from linearity in the final sections of the relationship *i_pa_* = f(*v*^1/2^) in MeCN. Another reason for the better quality of kinetics in MeCN was the higher DN value, which increased the association in this solvent, which caused the appearance of larger gaps in the *poly*[Ni(salcn)] films after the electropolymerization process, resulting from the transport of larger ions.

## Figures and Tables

**Table 1:** Molar mass (*M*/g·mol^−1^) obtained from the slopes of Δ*m* = f(*Q*) plots and molar participations of ions and solvent (*n* + *n_s_*) in oxidation of *poly*[Ni(salcn)] (obtained as a result of 1 electropolymerization cycle) in various electrolyte solutions (10^−1^ mol·dm^−3^), in CH_2_Cl_2_ and MeCN.

		PF_6_^−^				ClO_4_^−^				BF_4_^−^			
		I		II		I		II		I		II	
	*v*/V·s^−1^	*M*	*n + n_s_*	*M*	*n + n_s_*	*M*	*n + n_s_*	*M*	*n + n_s_*	*M*	*n + n_s_*	*M*	*n + n_s_*
CH_2_Cl_2_	0.05	97	0.7	145	1 + 0.0	65	0.6	92	0.9 + 0	43	0.5	63	0.7
	0.01	97	0.7	164	1 + 0.2	65	0.6	116	1 + 0.2	48	0.5	63	0.7
	0.05	116	0.8	154	1 + 0.1	74	0.7	99	1 + 0.0	41	0.5	73	0.8
	after red. 40 s, 0 V												
MeCN	0.05	145	1.0	309	1 + 4.0	97	1.0	241	1 + 3.6	87	1.0	154	1 + 1.6
	0.01	145	1.0	309	1 + 4.0	96	1.0	251	1 + 3.7	87	1.0	157	1 + 1.6

**Table 2:** Molar mass (*M*/g·mol^−1^) obtained from the slopes of Δ*m* = f(*Q*) plots and molar participations of ions and solvent (*n* + *n_s_*) in oxidation of *poly*[Ni(salcn(Me))] and *poly*[Ni(salcn(Bu))] (obtained as a result of 1 electropolymerization cycle) in various electrolyte solutions (10^−1^ mol·dm^−3^), in CH_2_Cl_2_.

		PF_6_^−^				ClO_4_^−^				BF_4_^−^			
		I		II		I		II		I		II	
Polymer	*v*/V·s^−^^1^	*M*	*n + n_s_*	*M*	*n + n_s_*	*M*	*n + n_s_*	*M*	*n + n_s_*	*M*	*n + n_s_*	*M*	*n + n_s_*
*Poly*													
[Ni(salcn	0.05	347	1 + 2.4	164	1 + 0.2	304	1 + 2.4	116	1 + 0.2	270	1 + 2.2	37	0.4
(Me))]	0.01	367	1 + 2.6	193	1 + 0.6	304	1 + 2.4	135	1 + 0.4	270	1 + 2.2	39	0.4
*Poly*													
[Ni(salcn	0.05	483	1 + 4.0	323	1 + 2.1	436	1 + 4.0	280	1 + 2.1	415	1 + 4.0	66	0.8
(Bu))]	0.01	484	1 + 4.0	318	1 + 2.0	443	1 + 4.0	280	1 + 2.1	415	1 + 4.0	87	1.0

**Table 3:** Kinetic data for *poly*[Ni(salcn)] in various electrolyte solutions (10^−1^ mol·dm^−3^) and in various solvents.

10^8^ *cD*^1/2^/mol·cm^−2^·s^−1/2^													
		ClO_4_^−^		PF_6_^−^/ClO_4_^− b^		BF_4_^−^				PF_6_^−^/BF_4_^− c^		PF_6_^−^	
	*n* ^a^	*cD_a_* ^1/2^	*cD_c_* ^1/2^	*cD_a_* ^1/2^	*cD_c_* ^1/2^	*cD_a_* ^1/2^	*cD_a_* ^1/2^	*cD_c_* ^1/2^	*cD_c_* ^1/2^	*cD_a_* ^1/2^	*cD_c_* ^1/2^	*cD_a_* ^1/2^	*cD_c_* ^1/2^
		II	I	II	I	I	II	I	II	II	I	II	I
CH_2_Cl_2_	3	0.163	0.154	0.213	0.197	0.144	-	-	-	0.225	-	0.184 ^d^	0.054 ^d^
	5	0.173	0.156	0.222	0.202	0.152	-	-	-	0.232	-	0.192 ^d^	0.060 ^d^
	10	0.187	0.140	0.239	0.206	0.169	-	-	-	0.253	-	0.207 ^d^	0.063 ^d^
	15	0.183	0.136	0.234	0.199	0.166	-	-	-	0.249	-	0.205 ^d^	0.059 ^d^
	20	0.180	0.132	0.226	0.184	0.162	-	-	-	0.240	-	0.200 ^d^	0.057 ^d^
MeCN	3	0.213	0.180	-	-	0.204	0.234	0.101	0.674	-	-	0.237	0.202
	5	0.227	0.191	-	-	0.213	0.247	0.106	0.682	-	-	0.254	0.215
	10	0.243	0.196	-	-	0.237	0.269	0.118	0.777	-	-	0.273	0.224
	15	0.254	0.207	-	-	0.246	0.280	0.121	0.786	-	-	0.286	0.244
	20	0.260	0.211	-	-	0.252	0.285	0.124	0.789	-	-	0.294	0.246

^a^ numbers of polymerization cycles; ^b^ electropolymerization in TBAPF_6_, redox switching in TBAClO_4_; ^c^ electropolymerization in TBAPF_6_, switching redox in TBABF_4_; ^d^ data from [38].

**Table 4:** Kinetic data for *poly*[Ni(salcn(Me))] in various electrolyte solutions (10^−1^ mol·dm^−3^) in CH_2_Cl_2_.

10^8^ *cD*^1/2/^mol·cm^−2^·s^−1/2^										
	PF_6_^−^		ClO_4_^−^		PF_6_^−^/ClO_4_^− b^		BF_4_^−^		PF_6_^−^/BF_4_^− c^	
*n* ^a^	*cD_a_* ^1/2^	*cD_c_* ^1/2^	*cD_a_* ^1/2^	*cD_c_* ^1/2^	*cD_a_* ^1/2^	*cD_c_* ^1/2^	*cD_a_* ^1/2^	*cD_c_* ^1/2^	*cD_a_* ^1/2^	*cD_c_* ^1/2^
	I	II	I	II	I	II	I	II	I	II
5	0.808 ^d^	0.394 ^d^	0.624	0.489	1.25	0.982	0.520	0.481	1.56	1.43
10	1.49 ^d^	0.629 ^d^	1.13	0.762	2.25	1.59	0.937	0.856	2.82	2.51
15	1.97 ^d^	1.03 ^d^	1.49	0.942	2.94	2.10	1.29	1.16	3.82	3.45
20	2.34 ^d^	1.22 ^d^	1.72	1.10	3.56	2.42	1.57	1.31	4.45	3.90
25	2.53 ^d^	1.37 ^d^	1.99	1.24	3.89	2.61	1.67	1.33	4.90	4.16
30	2.76 ^d^	1.43 ^d^	2.13	1.32	4.17	2.83	1.85	1.46	5.19	4.31

^a^ numbers of polymerization cycles; ^b^ electropolymerization in TBAPF_6_, redox switching in TBAClO_4_; ^c^ electropolymerization in TBAPF_6_, switching redox in TBABF_4_; ^d^ data from [38].

**Table 5:** Kinetic data for *poly*[Ni(salcn(Bu))] in various electrolyte solutions (10^−1^ mol·dm^−3^) in CH_2_Cl_2_.

10^8^ *cD*^1/2*/*^mol·cm^−2^·s^−1/2^								
	ClO_4_^−^				PF_6_^−^/ClO_4_^− b^			
*n* ^a^	*cD_a_* ^1/2^	*cD_a_* ^1/2^	*cD_c_* ^1/2^	*cD_c_* ^1/2^	*cD_a_* ^1/2^	*cD_a_* ^1/2^	*cD_c_* ^1/2^	*cD_c_* ^1/2^
	I	II	I	II	I	II	I	II
10	1.09	1.43	1.39	0.960	2.60	3.32	3.41	2.32
15	1.67	2.15	2.06	1.49	3.96	5.03	5.18	3.52
20	1.97	2.54	2.46	1.74	4.73	5.96	6.13	4.17
25	2.59	3.21	3.10	2.38	6.20	7.75	7.94	5.48
30	3.02	3.69	3.54	3.09	7.27	9.01	9.25	6.43
	**BF_4_^−^**				**PF_6_^−^/BF_4_^−^** **^c^**			
10	0.856	1.29	0.288	-	3.15	4.81	1.09	-
15	1.34	1.99	0.446	-	5.12	7.63	1.74	-
20	1.55	2.27	0.509	-	5.90	8.74	2.07	-
25	2.06	3.01	0.662	-	7.71	11.5	2.55	-
30	2.42	3.53	0.770	-	8.94	13.3	2.94	-
	**PF_6_^−^^d^**							
10	1.61	1.71	1.74	1.22				
15	2.51	2.63	2.68	1.85				
20	2.94	3.07	3.21	2.12				
25	3.95	3.87	3.85	2.74				
30	4.57	4.46	4.49	3.36				

^a^ numbers of polymerization cycles; ^b^ electropolymerization in TBAPF_6_, redox switching in TBAClO_4_; ^c^ electropolymerization in TBAPF_6_, switching redox in TBABF_4_; ^d^ data from [38].

## Data Availability

Data is contained within the article.

## Supplementary Materials

The following supporting information can be downloaded at: https://www.mdpi.com/article/10.3390/molecules27061812/s1, Figure S1. FTIR ATR spectra of poly[Ni(salcn(Me))] electrosynthezed in TBABF4/CH2Cl2 (*v* = 0.05 V·s^−1^, 10 scans, vs. Ag/AgCl), (A) neutral, (B) oxidized at the anode peak potential, Epa = 0.6 V. Figure S2. Poly[Ni(salcn)] (1 electropolymerization cycle) in TBAPF6/CH2Cl2, Pt/quarzglas, *v* = 0.05 V⋅s^−1^, vs. Ag/AgCl; (A) cyclic voltammogram; (B) plots Δ*m* vs. Q for anodic process; grey parts of the curve—trend lines for the Δ*m* = f(*Q*) relationship. Figure S3. Poly[Ni(salcn)] (1 electropolymerization cycle) in TBABF4/CH2Cl2, Pt/quarzglas, *v* = 0.05 V⋅s^−1^, vs. Ag/AgCl; (A) cyclic voltammogram; (B) plots Δ*m* vs. Q for anodic process; grey parts of the curve—trend lines for the Δ*m* = f(*Q*) relationship. Figure S4. Poly[Ni(salcn(Me))] (1 electropolymerization cycle) in TBAPF6/CH2Cl2, Pt/quarzglas, *v* = 0.05 V⋅s^−1^, vs. Ag/AgCl; (A) cyclic voltammogram; (B) plot Δ*m* vs. Q for anodic process; grey parts of the curve—trend lines for the Δ*m* = f(*Q*) relationship. Figure S5. Poly[Ni(salcn(Me))] (1 electropolymerization cycle) in TBABF4/CH2Cl2, Pt/quarzglas, *v* = 0.05 V⋅s^−1^, vs. Ag/AgCl; (A) cyclic voltammogram; (B) plot Δ*m* vs. Q for anodic process; grey parts of the curve—trend lines for the Δ*m* = f(*Q*) relationship. Figure S6. Poly[Ni(salcn(Bu))] (1 electropolymerization cycle) in TBAPF6/CH2Cl2, Pt/quarzglas, *v* = 0.05 V⋅s^−1^, vs. Ag/AgCl; (A) cyclic voltammogram; (B) plot Δ*m* vs. Q for anodic process; grey parts of the curve—trend lines for the Δ*m* = f(*Q*) relationship. Figure S7. Poly[Ni(salcn(Bu))] (1 electropolymerization cycle) in TBABF4/CH2Cl2, Pt/quarzglas, *v* = 0.05 V⋅s^−1^, vs. Ag/AgCl; (A) cyclic voltammogram; (B) plot Δ*m* vs. Q for anodic process; grey parts of the curve—trend lines for the Δ*m* = f(*Q*) relationship. Figure S8. Plots Δ*m* vs. Q for poly[Ni(salcn)] for anodic process (1 electropolymerization cycle) in TBAPF6H/CH2Cl2, Pt/quarzglas, *v* = 0.01 V⋅s^−1^, vs. Ag/AgCl; grey parts of the curve—trend lines for the Δ*m* = f(*Q*) relationship. Figure S9. Plots Δ*m* vs. Q for poly[Ni(salcn)] for anodic process (1 electropolymerization cycle) in TBABF4/CH2Cl2, Pt/quarzglas, *v* = 0.01 V⋅s^−1^, vs. Ag/AgCl; grey parts of the curve—trend lines for the Δ*m* = f(*Q*) relationship. Figure S10. Plots Δ*m* vs. Q for poly[Ni(salcn)] for anodic process (1 electropolymerization cycle) in TBAPF6/CH2Cl2, Pt/quarzglas, *v* = 0.05 V⋅s^−1^, after reduction 40 s at 0 V, vs. Ag/AgCl; grey parts of the curve—trend lines for the Δ*m* = f(*Q*) relationship. Figure S11. Plots Δ*m* vs. Q for poly[Ni(salcn)] for anodic process (1 electropolymerization cycle) in TBABF4/CH2Cl2, Pt/quarzglas, *v* = 0.05 V⋅s^−1^, after reduction 40 s at 0 V, vs. Ag/AgCl; grey parts of the curve—trend lines for the Δ*m* = f(*Q*) relationship. Figure S12. Poly[Ni(salcn)] (1 electropolymerization cycle) in TBAPF6/MeCN, Pt/quarzglas, *v* = 0.05 V⋅s^−1^, vs. Ag/AgCl; (A) cyclic voltammogram; (B) plot Δ*m* vs. Q for anodic process; grey parts of the curve—trend lines for the Δ*m* = f(*Q*) relationship. Figure S13. Poly[Ni(salcn)] (1 electropolymerization cycle) in TBABF4/MeCN, Pt/quarzglas, *v* = 0.05 V⋅s^−1^, vs. Ag/AgCl; (A) cyclic voltammogram; (B) plot Δ*m* vs. Q for anodic process; grey parts of the curve—trend lines for the Δ*m* = f(*Q*) relationship. Figure S14. Poly[Ni(salcn)] (10 electropolymerization cycles) in TBAClO4/CH2Cl2, Pt, *v* = 0.03–1 V⋅s^−1^, vs. Ag/AgCl; (A) the lower part—cyclic voltammograms; the upper part—voltammograms for *v* = 0.01–0.09 V⋅s^−1^; (B) plots ip vs. v1/2 for oxidation and reduction processes. Figure S15. Poly[Ni(salcn)] (3 electropolymerization cycles) in TBABF4/CH2Cl2, Pt, *v* = 0.02–1 V⋅s^−1^, vs. Ag/AgCl; (A) the lower part—cyclic voltammograms; the upper part—voltammograms for *v* = 0.01–0.15 V⋅s^−1^; (B) plots ip vs. v1/2 for oxidation process. Figure S16. Cyclic voltammogram of poly[Ni(salcn(Me))] (10 electropolymerization cycles) in TBAClO4/CH2Cl2, Pt, *v* = 0.05 V⋅s^−1^, vs. Ag/AgCl. Figure S17. Poly[Ni(salcn(Me))] (15 electropolymerization cycles) in TBABF4/CH2Cl2, Pt, *v* = 0.01–1 V⋅s^−1^, vs. Ag/AgCl; (A) cyclic voltammograms for one-step process, peak notations were used adequate to peak designation in a two-step process, see Figure 3; (B) plots ip vs. v1/2 for oxidation and reduction processes. Figure S18. Poly[Ni(salcn(Bu))] (25 electropolymerization cycles) in TBAClO4/CH2Cl2, Pt, *v* = 0.01–1 V⋅s^−1^, vs. Ag/AgCl; (A) cyclic voltammograms; (B) plots ip vs. v1/2 for Ist oxidation step of the process and IInd reduction step; (C) plots ip vs. v1/2 for IInd oxidation step of the process and Ist reduction step. Figure S19. Poly[Ni(salcn(Bu))] (25 electropolymerization cycles) in TBABF4/CH2Cl2, Pt, *v* = 0.02–1 V⋅s^−1^, vs. Ag/AgCl; (A) cyclic voltammograms; (B) plots ip vs. v1/2 for Ist oxidation step of the process; (C) plots ip vs. v1/2 for IInd oxidation step of the process and Ist reduction step. Figure S20. Cyclic voltammograms of poly[Ni(salcn)], 1st–10th cycle, in TBABF4/CH2Cl2, Pt, *v* = 0.05 V⋅s^−1^, vs. Ag/AgCl; after 5 electropolymerization cycles. Figure S21. Cyclic voltammograms of poly[Ni(salcn(Bu))], 1st–10th cycle, in TBABF4/CH2Cl2, Pt, *v* = 0.05 V⋅s^−1^, vs. Ag/AgCl; after 5 electropolymerization cycles. Figure S22. Poly[Ni(salcn)] (3 electropolymerization cycles) in TBAClO4/MeCN, Pt, *v* = 0.01–1 V⋅s^−1^, vs. Ag/AgCl; (A) the lower part—cyclic voltammograms, the upper part—voltammograms for *v* = 0.01–0.25 V⋅s^−1^; (B) plots ip vs. v1/2 for oxidation and reduction processes. Figure S23. Poly[Ni(salcn)] (3 electropolymerization cycles) in TBABF4/MeCN, Pt, *v* = 0.02–1 V⋅s^−1^, vs. Ag/AgCl; (A) cyclic voltammograms; (B) plots ip vs. v1/2 for Ist oxidation step of the process and IInd reduction step; (C) plots ip vs. v1/2 for IInd oxidation step of the process and Ist reduction step.

## Sample Availability

Samples of the compounds [Ni(salcn)], [Ni(salcn(Me))] and [Ni(salcn(Bu))] are available from the authors.
