# Supplementary material for: The Influence of Electrolyte Type on Kinetics of Redox Processes in the Polymer Films of Ni(II) Salen-Type Complexes
Source: Molecules. 2022 Mar 10;27(6):1812. doi: 10.3390/molecules27061812 (PMC8954381; doi:10.3390/molecules27061812)
Supplement: Supplementary file 1 [file molecules-27-01812-s001.zip › molecules-1583602-supplementary.pdf]

Supplementary materials

The influence of electrolyte type on kinetics of redox processes in the polymer films of Ni(II) *salen* type complexes

D. Tomczyk*[1], P. Seliger[1], W. Bukowski[2], K. Bester[2]

Figures

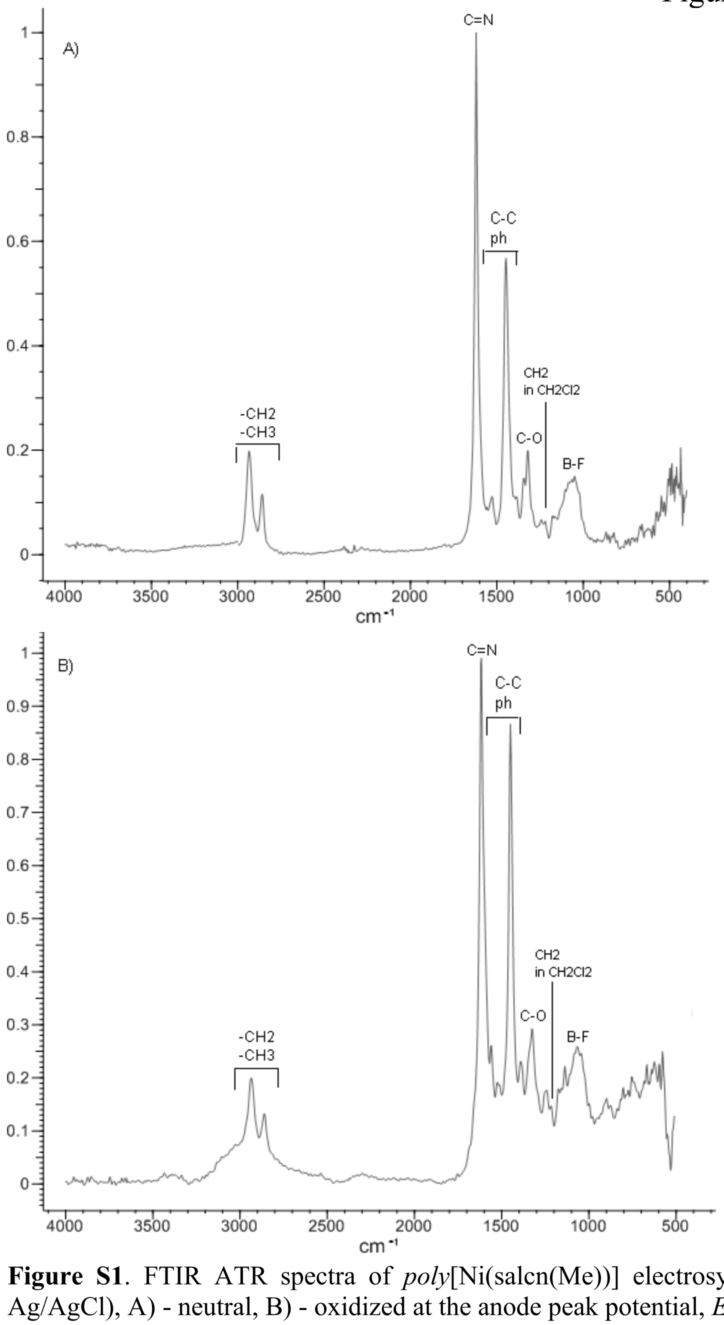

Figure S1. FTIR ATR spectra of *poly*[Ni(salcn(Me))] electrosynthesized in $TBABF_4/CH_2Cl_2$ (v = 0.05 $V \cdot s^{-1}$, 10 scans, vs. Ag/AgCl), A) - neutral, B) - oxidized at the anode peak potential, E_{pa} = 0.6 V.

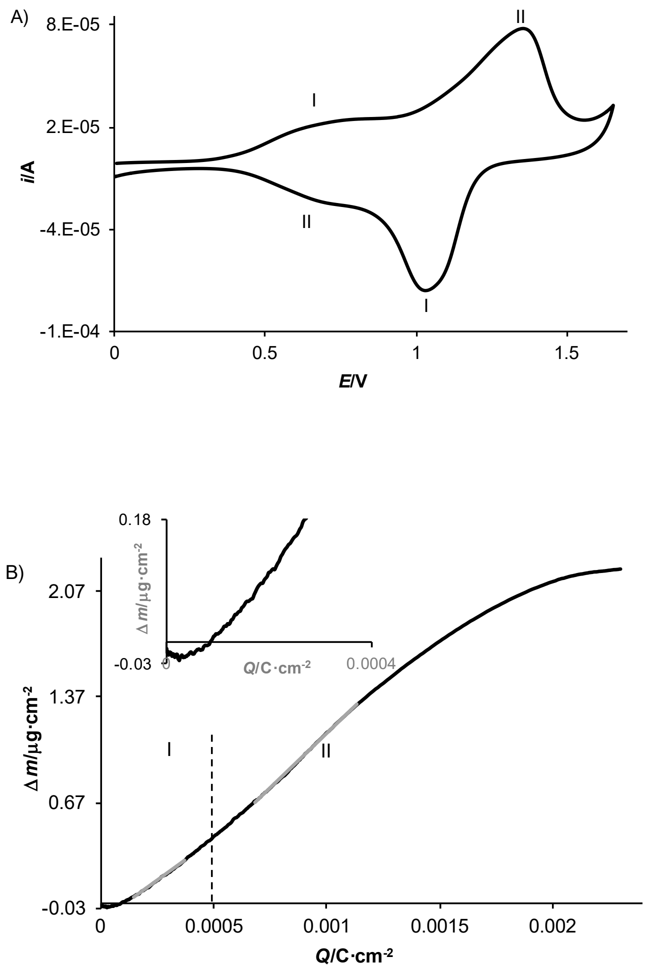

Figure S2. *Poly*[Ni(salcn)] (1 electropolymerization cycle) in $TBAPF_6/CH_2Cl_2$, Pt/quarzglas , $v = 0.05$ V·s^{-1}, vs. Ag/AgCl; A) cyclic voltammogram; B) plots Δm vs Q for anodic process; grey parts of the curve - trend lines for the $\Delta m = f(Q)$ relationship.

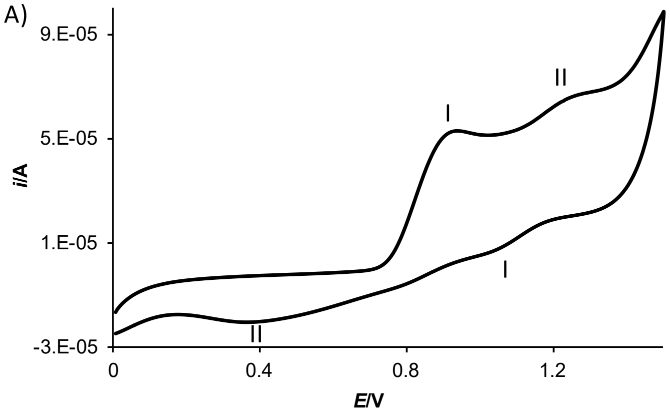

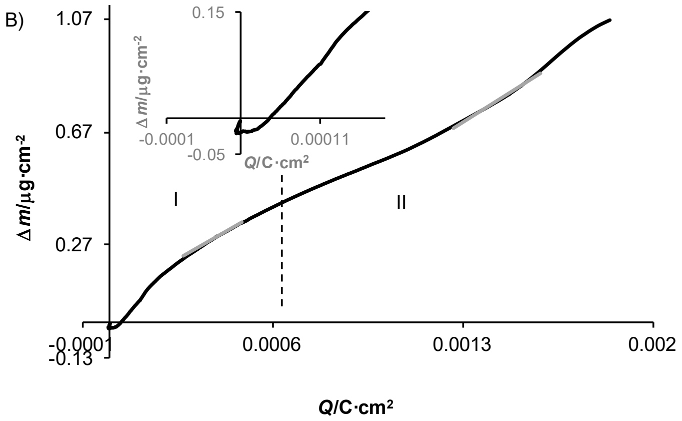

Figure S3. *Poly*[Ni(salcn)] (1 electropolymerization cycle) in $TBABF_4/CH_2Cl_2$, Pt/quarzglas , $v = 0.05$ V·s^{-1}, vs. Ag/AgCl; A) cyclic voltammogram; B) plots Δ*m* vs *Q* for anodic process; grey parts of the curve - trend lines for the Δ*m* = f(*Q)* relationship.

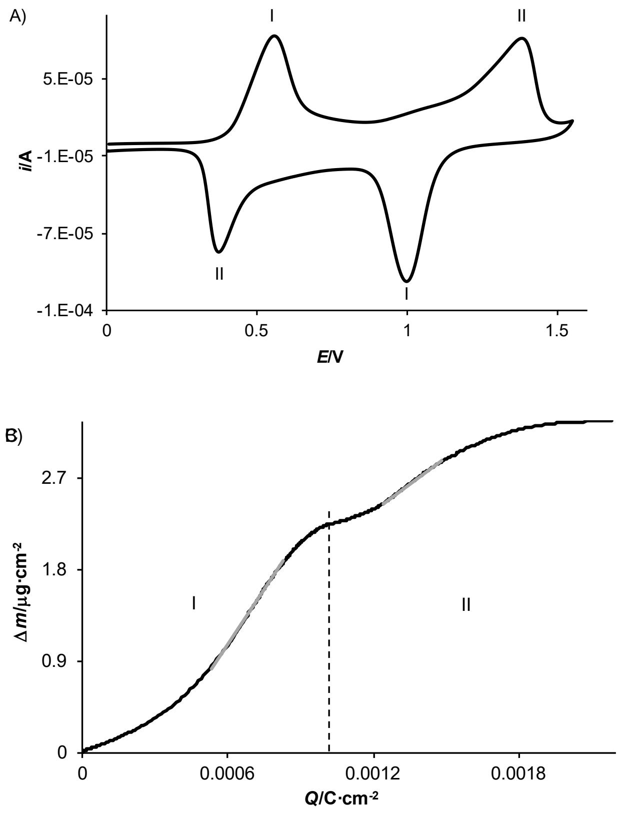

Figure S4. *Poly*[Ni(salcn(Me))] (1 electropolymerization cycle) in $TBAPF_6/CH_2Cl_2$, Pt/quarzglas , $v = 0.05$ V·s^{-1}, vs. Ag/AgCl; A) cyclic voltammogram; B) plot Δ*m* vs *Q* for oxidation process; grey parts of the curve - trend lines for the Δ*m* = f(*Q)* relationship.

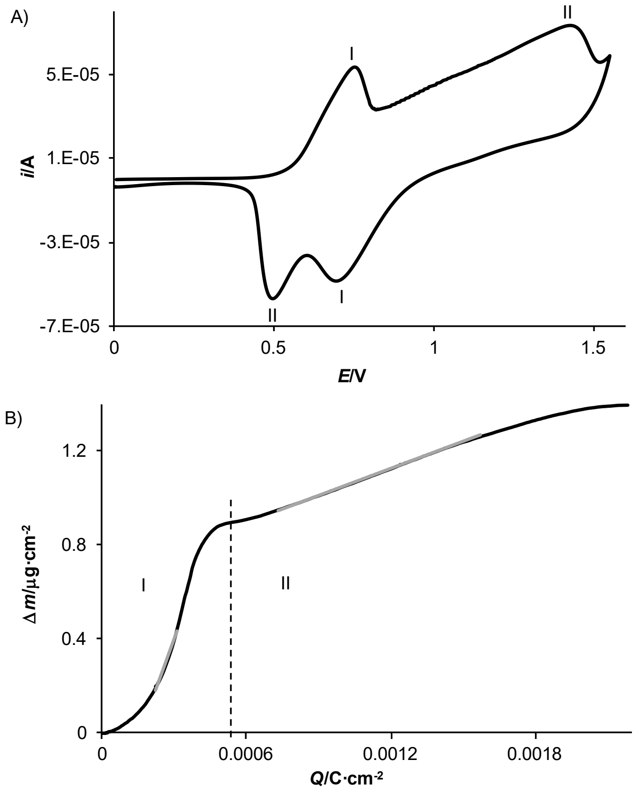

Figure S5. *Poly*[Ni(salcn(Me))] (1 electropolymerization cycle) in $TBABF_4/CH_2Cl_2$, Pt/quarzglas , $v = 0.05$ $V{\cdot}s^{-1}$, vs. Ag/AgCl; A) cyclic voltammogram; B) plot Δ*m* vs *Q* for anodic process; grey parts of the curve - trend lines for the Δ*m* = f(*Q)* relationship.

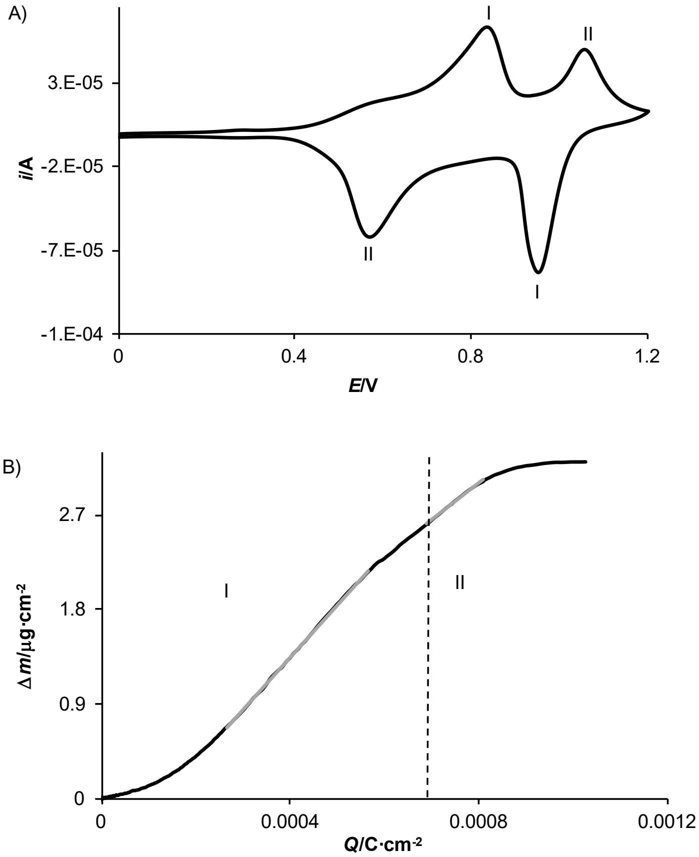

Figure S6. *Poly*[Ni(salcn(Bu))] (1 electropolymerization cycle) in TBAPF$_6$/CH$_2$Cl$_2$, Pt/quarzglas , v = 0.05 V·s^{-1}, vs. Ag/AgCl; A) cyclic voltammogram; B) plot Δ*m* vs *Q* for anodic process; grey parts of the curve - trend lines for the Δ*m* = f(*Q)* relationship.

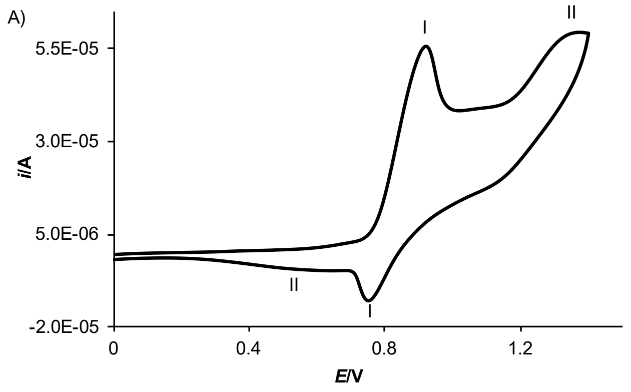

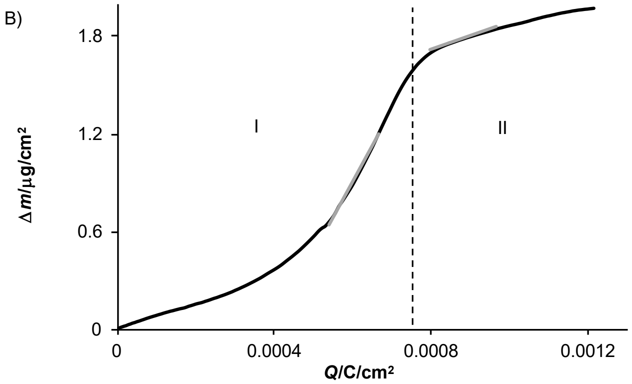

Figure S7. *Poly*[Ni(salcn(Bu))] (1 electropolymerization cycle) in $TBABF_4/CH_2Cl_2$, Pt/quarzglas , $v = 0.05$ V·s^{-1}, vs. Ag/AgCl; A) cyclic voltammogram; B) plot Δm vs Q for anodic process; grey parts of the curve - trend lines for the $\Delta m = f(Q)$ relationship.

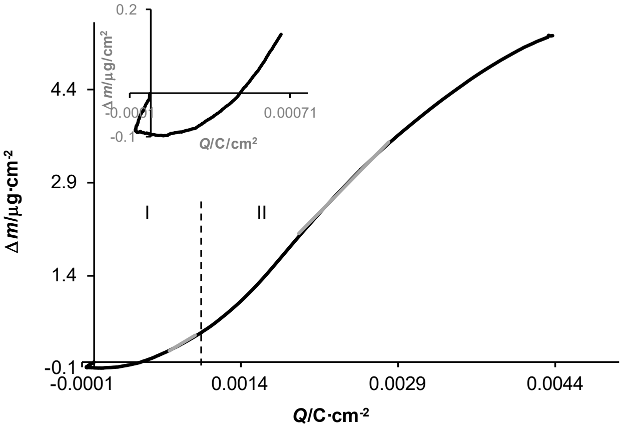

Figure S8. Plots Δm vs Q for *poly*[Ni(salcn)] for anodic process (1 electropolymerization cycle) in $TBAPF_6/CH_2Cl_2$, Pt/quarzglas , $v = 0.01$ V·s^{-1}, vs. Ag/AgCl; grey parts of the curve - trend lines for the $\Delta m = f(Q)$ relationship.

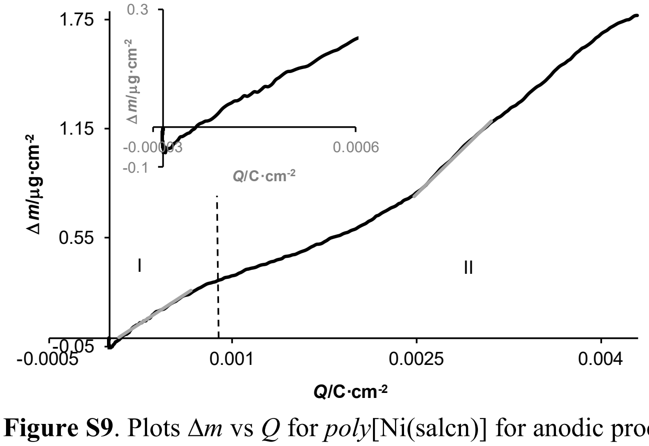

Figure S9. Plots Δm vs Q for *poly*[Ni(salcn)] for anodic process (1 electropolymerization cycle) in $TBABF_4/CH_2Cl_2$, Pt/quarzglas , $v = 0.01$ V·s^{-1}, vs. Ag/AgCl; grey parts of the curve - trend lines for the $\Delta m = f(Q)$ relationship.

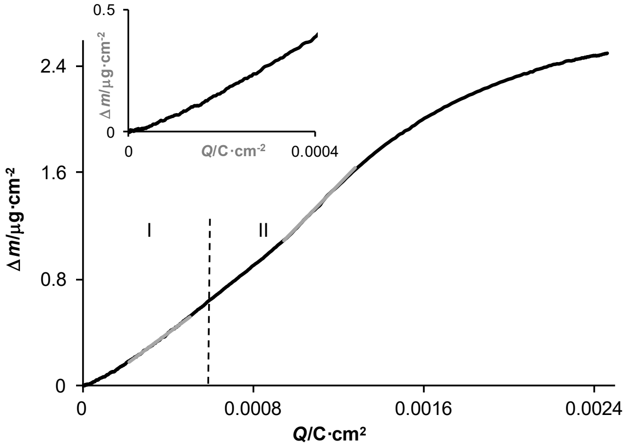

Figure S10. Plots Δm vs Q for *poly*[Ni(salcn)] for anodic process (1 electropolymerization cycle) in $TBAPF_6/CH_2Cl_2$, Pt/quarzglas , $v = 0.05$ V·s^{-1}, after reduction 40 s at 0 V, vs. Ag/AgCl; grey parts of the curve - trend lines for the Δm = f(Q) relationship.

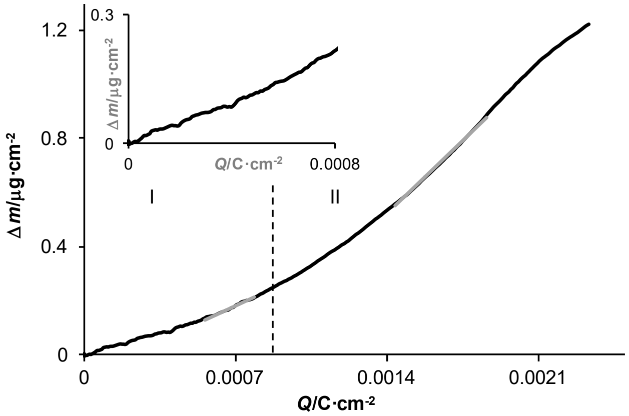

Figure S11. Plots Δm vs Q for *poly*[Ni(salcn)] for anodic process (1 electropolymerization cycle) in $TBABF_4/CH_2Cl_2$, Pt/quarzglas , $v = 0.05$ V·s^{-1}, after reduction 40 s at 0 V, vs. Ag/AgCl; grey parts of the curve - trend lines for the Δm = f(Q) relationship.

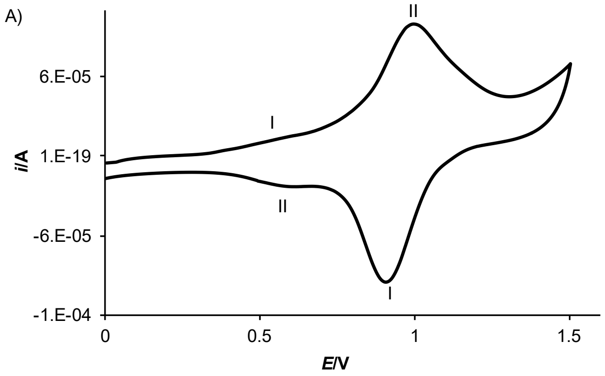

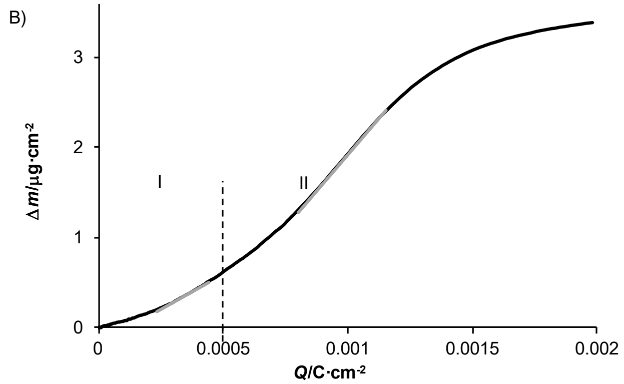

Figure S12. Figure S12. *Poly*[Ni(salcn)] (1 electropolymerization cycle) in $TBAPF_6$/MeCN, Pt/quarzglas , $v = 0.05$ V·s^{-1}, vs. Ag/AgCl; A) cyclic voltammogram; B) plot Δ*m* vs *Q* for anodic process; grey parts of the curve - trend lines for the Δ*m* = f(*Q)* relationship.

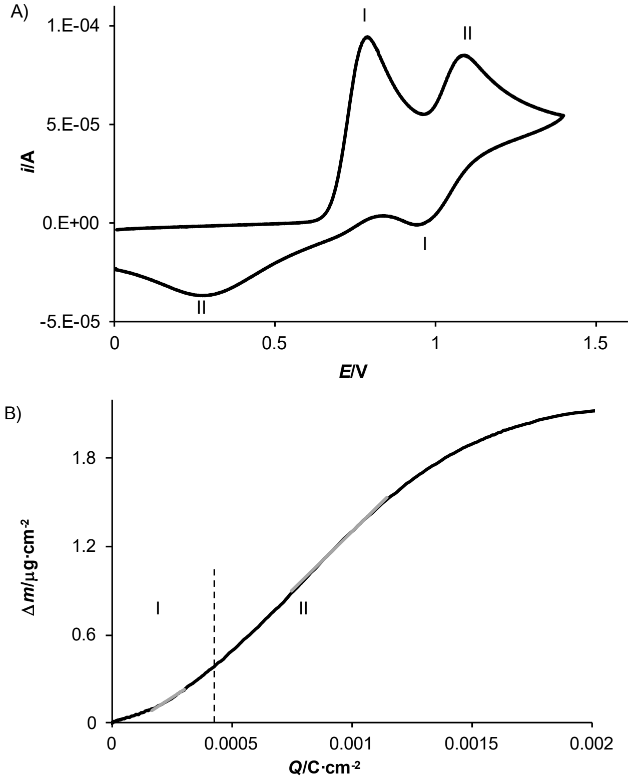

Figure S13. *Poly*[Ni(salcn)] (1 electropolymerization cycle) in $TBABF_4$/MeCN, Pt/quarzglas , $v = 0.05$ V·s^{-1}, vs. Ag/AgCl; A) cyclic voltammogram; B) plot Δ*m* vs *Q* for anodic process; grey parts of the curve - trend lines for the Δ*m* = f(*Q)* relationship.

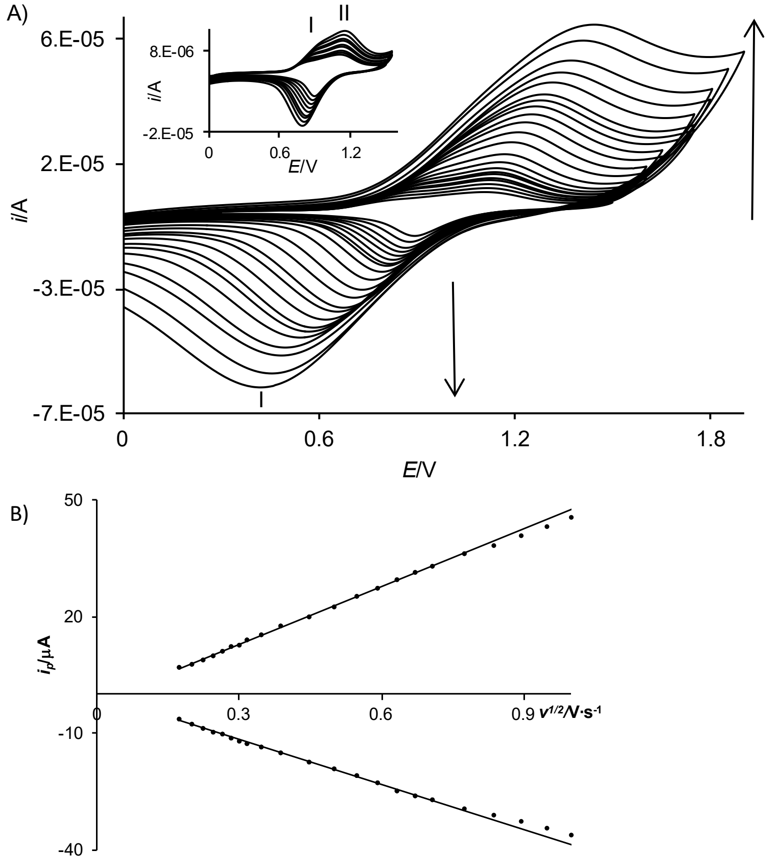

Figure S14. *Poly*[Ni(salcn)] (10 electropolymerization cycles) in $TBAClO_4/CH_2Cl_2$, Pt, $v = 0.03 - 1$ V·s^{-1}, vs. Ag/AgCl; A) the lower part - cyclic voltammograms; the upper part – voltammograms for $v = 0.01 – 0.09$ V·s^{-1}; B) plots i_p vs $v^{1/2}$ for oxidation and reduction processes.

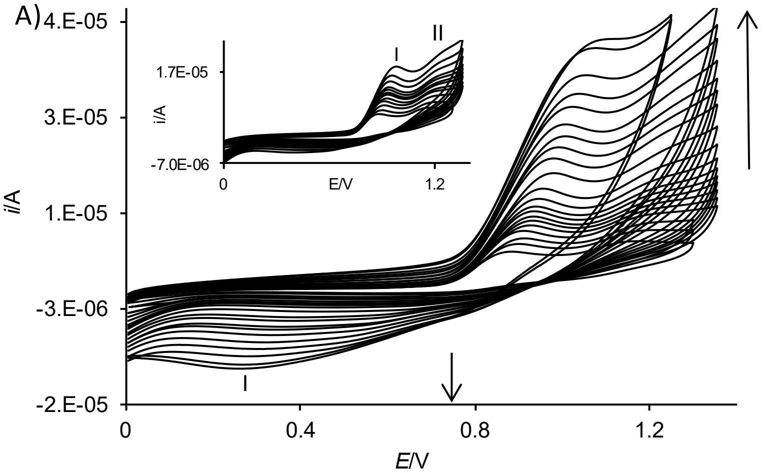

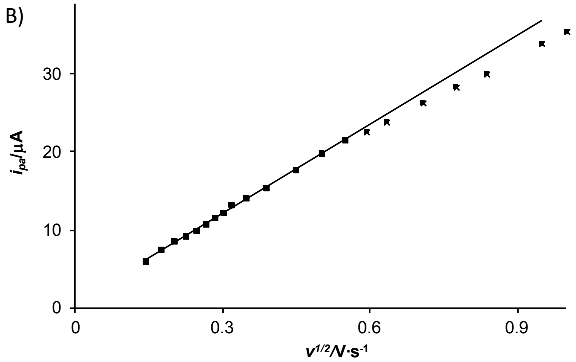

Figure S15. *Poly*[Ni(salcn)] (3 electropolymerization cycles) in $TBABF_4/CH_2Cl_2$, Pt, $v = 0.02$ - 1 $V\cdot s^{-1}$, vs. Ag/AgCl; A) the lower part - cyclic voltammograms; the upper part – voltammograms for $v = 0.01 – 0.15$ $V\cdot s^{-1}$; B) plot i_{pa} vs $v^{1/2}$ for oxidation process.

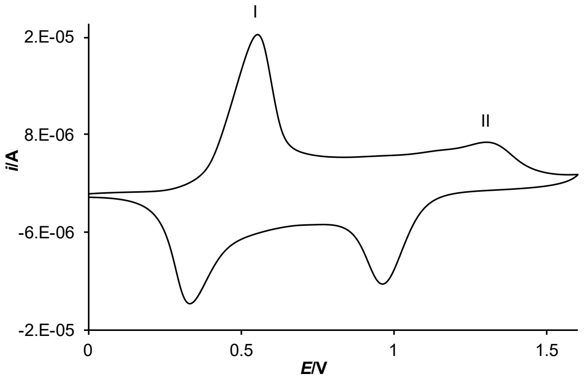

Figure S16. Cyclic voltammogram of *poly*[Ni(salcn(Me))] (10 electropolymerization cycles) in $TBAClO_4/CH_2Cl_2$, Pt, $v = 0.05$ $V\cdot s^{-1}$, vs. Ag/AgCl.

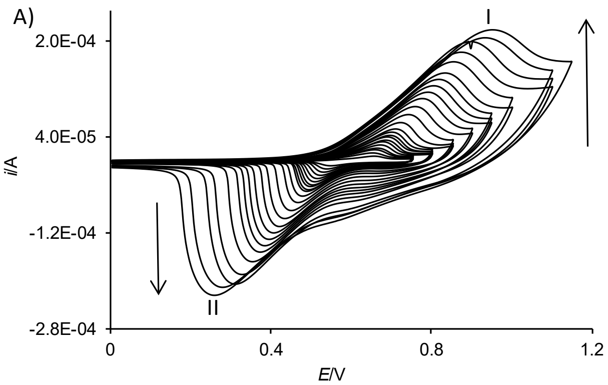

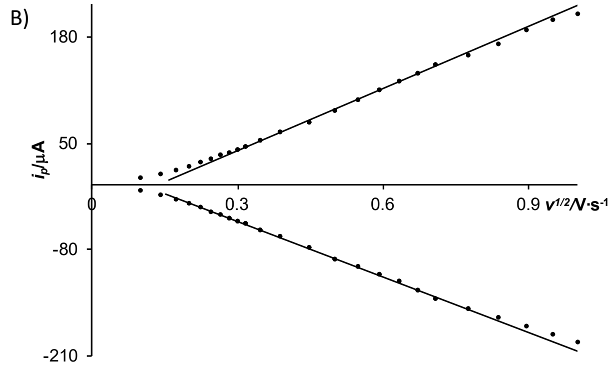

Figure S17. *Poly*[Ni(salcn(Me))] (15 electropolymerization cycles) in $TBABF_4/CH_2Cl_2$, Pt, $v = 0.01$ - 1 V·s^{-1}, vs. Ag/AgCl; A) cyclic voltammograms for one-step process, peak notations were used adequate to peak designation in a two-step process, see Figure 3; B) plots i_{pa} vs $v^{1/2}$ for oxidation and reduction processes.

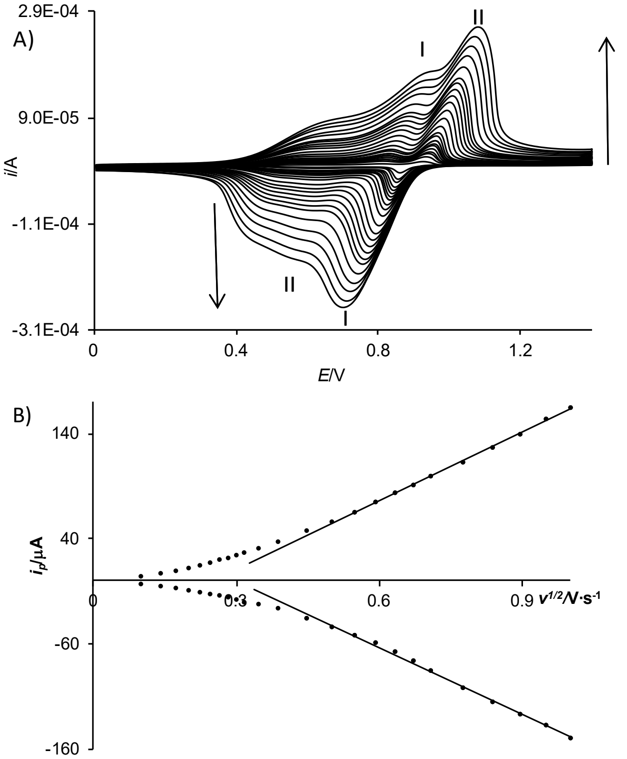

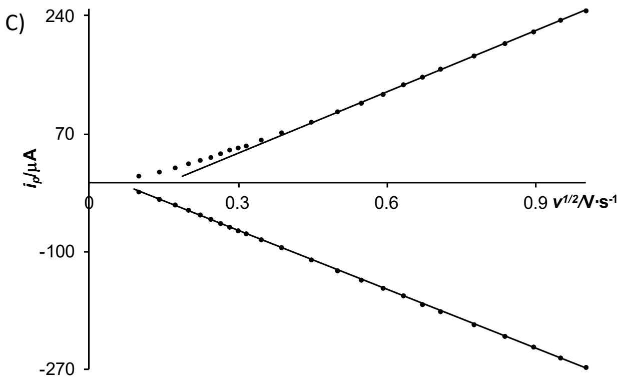

Figure S18. *Poly*[Ni(salcn(Bu))] (25 electropolymerization cycles) in $TBAClO_4/CH_2Cl_2$, Pt, $v = 0.01$ - 1 V·s^{-1}, vs. Ag/AgCl; A) cyclic voltammograms; B) plots i_p vs $v^{1/2}$ for Ist oxidation step of the process and IInd reduction step*;* C) plots i_p vs $v^{1/2}$ for IInd oxidation step of the process and Ist reduction step.

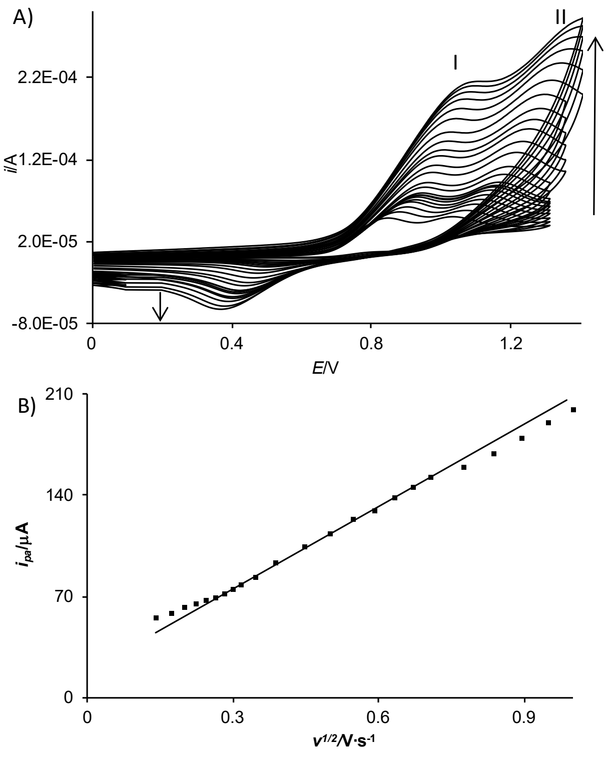

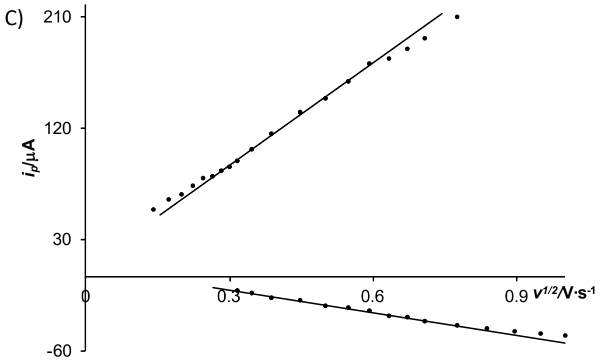

Figure S19. *Poly*[Ni(salcn(Bu))] (25 electropolymerization cycles) in $TBABF_4/CH_2Cl_2$, Pt, $v = 0.02 - 1$ V·s^{-1}, vs. Ag/AgCl; A) cyclic voltammograms; B) plot i_p vs $v^{1/2}$ for Ist oxidation step of the process; C) plots i_p vs $v^{1/2}$ for IInd oxidation step of the process and Ist reduction step.

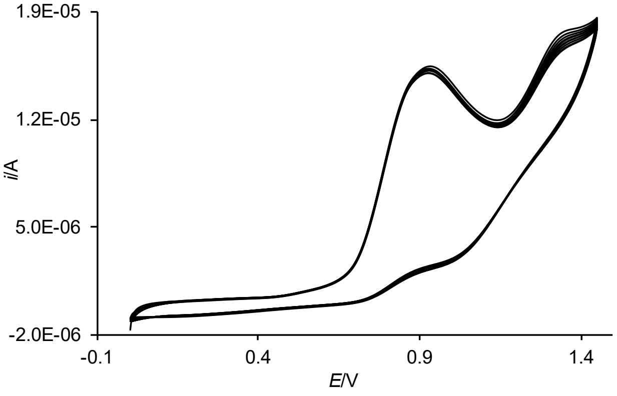

Figure S20. Cyclic voltammograms *of poly*[Ni(salcn)], 1st-10th cycle, in $TBABF_4/CH_2Cl_2$, Pt, $v = 0.05$ V·s^{-1}, vs. Ag/AgCl; after 5 electropolymerization cycles.

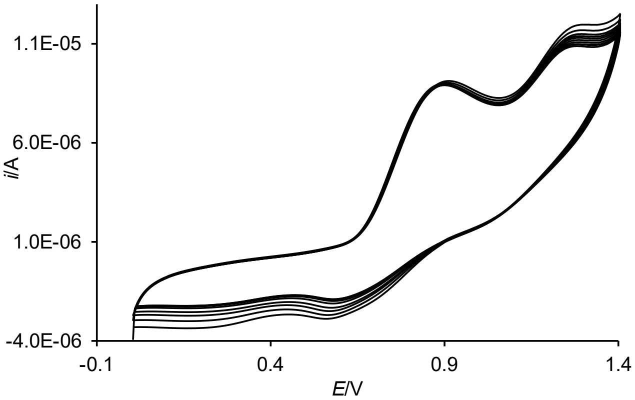

Figure S21. Cyclic voltammograms *of poly*[Ni(salcn(Bu))], 1st-10th cycle, in $TBABF_4/CH_2Cl_2$, Pt, $v = 0.05$ V·s^{-1}, vs. Ag/AgCl; after 5 electropolymerization cycles.

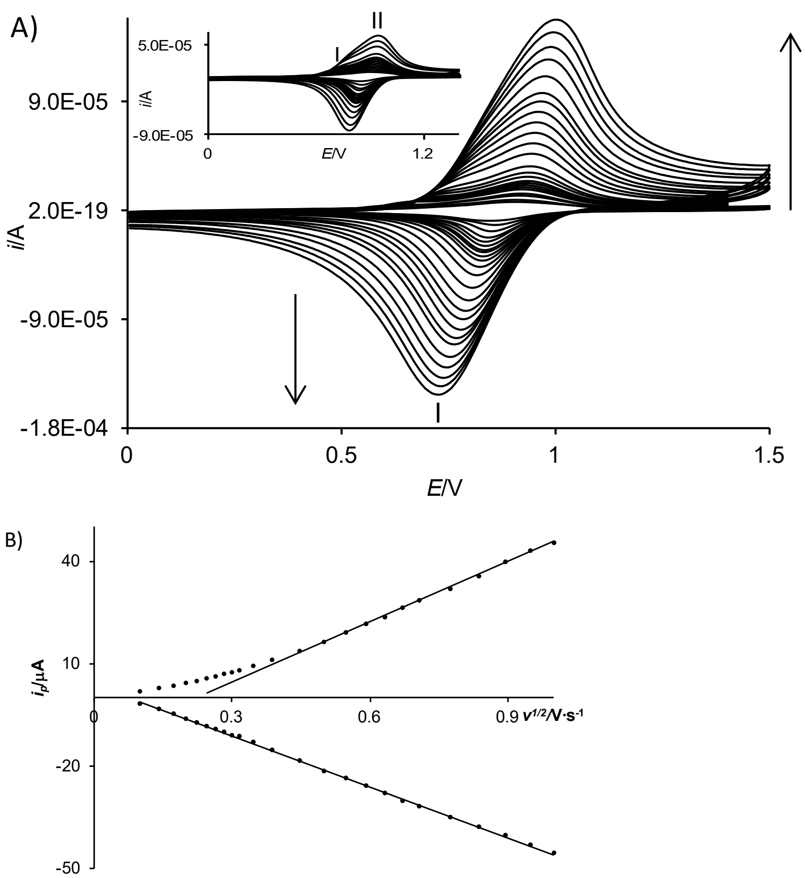

Figure S22. *Poly*[Ni(salcn)] (3 electropolymerization cycles) in $TBAClO_4$/MeCN, Pt, $v = 0.01$ - 1 V·s^{-1}, vs. Ag/AgCl; A) the lower part - cyclic voltammograms, the upper part – voltammograms for $v = 0.01 – 0.25$ V·s^{-1}; B) plots i_{pa} vs $v^{1/2}$ for oxidation and reduction processes.

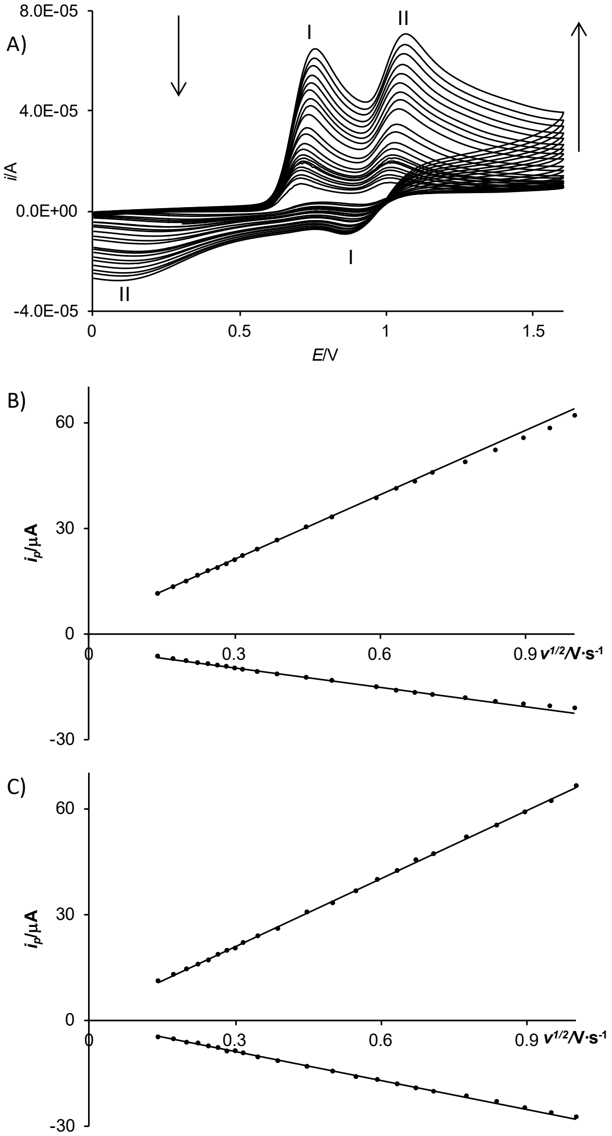

Figure S23. *Poly*[Ni(salcn)] (3 electropolymerization cycles) in $TBABF_4$/MeCN, Pt, $v = 0.02$ - 1 V·s^{-1}, vs. Ag/AgCl; A) cyclic voltammograms; B) plots i_p vs $v^{1/2}$ for Ist oxidation step of the process and IInd reduction step*;* C) plots i_p vs $v^{1/2}$ for IInd oxidation step of the process and Ist reduction step.
